# The Impact of Hidden Structure on Aggregate Disassembly by Molecular Chaperones

**DOI:** 10.3389/fmolb.2022.915307

**Published:** 2022-07-07

**Authors:** Daniel Shoup, Andrew Roth, Jason Puchalla, Hays S. Rye

**Affiliations:** ^1^ Department of Biochemistry and Biophysics, Texas A&M University, College Station, TX, United States; ^2^ Department of Physics, Princeton University, Princeton, NJ, United States

**Keywords:** protein disaggregase, molecular chaperone, single particle fluorescence, protein aggregation, protein aggregate detection

## Abstract

Protein aggregation, or the uncontrolled self-assembly of partially folded proteins, is an ever-present danger for living organisms. Unimpeded, protein aggregation can result in severe cellular dysfunction and disease. A group of proteins known as molecular chaperones is responsible for dismantling protein aggregates. However, how protein aggregates are recognized and disassembled remains poorly understood. Here we employ a single particle fluorescence technique known as Burst Analysis Spectroscopy (BAS), in combination with two structurally distinct aggregate types grown from the same starting protein, to examine the mechanism of chaperone-mediated protein disaggregation. Using the core bi-chaperone disaggregase system from *Escherichia coli* as a model, we demonstrate that, in contrast to prevailing models, the overall size of an aggregate particle has, at most, a minor influence on the progression of aggregate disassembly. Rather, we show that changes in internal structure, which have no observable impact on aggregate particle size or molecular chaperone binding, can dramatically limit the ability of the bi-chaperone system to take aggregates apart. In addition, these structural alterations progress with surprising speed, rendering aggregates resistant to disassembly within minutes. Thus, while protein aggregate structure is generally poorly defined and is often obscured by heterogeneous and complex particle distributions, it can have a determinative impact on the ability of cellular quality control systems to process protein aggregates.

## Introduction

The folding of proteins within the complex and concentrated interior of a cell frequently goes awry, resulting in mis-folding and aggregation (Ellis and Minton, 2006; Chiti and Dobson, 2017). Work on a variety of human diseases has shown that the incorrect folding and/or aggregation of important cellular proteins can lead to a wide variety of serious pathologies (Balch et al., 2008; Chiti and Dobson, 2017). The competition between folding, misfolding and aggregation spawned the evolution of specialized protein folding machines and quality control systems, which are arranged into interlocking networks of molecular chaperones, regulated proteases, conformational sensors and transcriptional feedback circuits (Hartl et al., 2011; Labbadia and Morimoto, 2015).

While proteostatic networks can be organized in a variety of ways, the core components are conserved across phylogeny, including several families of essential molecular chaperones (Rosenzweig et al., 2019; Balchin et al., 2020). Importantly, while molecular chaperones are generally required for facilitating protein folding and preventing aggregation, molecular chaperones are also necessary for recognizing and dismantling protein aggregates (Mogk et al., 2018; Fassler et al., 2021). The Hsp70 class of molecular chaperones and some of their associated cofactors (the Hsp40 targeting factors and nucleotide exchange factors or NEFs), play a central role in the response to aggregate formation in virtually all organisms (Mogk et al., 2018; Mayer and Gierasch, 2019; Kohler and Andréasson, 2020). Most bacteria, fungi and plants also employ a second important chaperone of the Hsp100 class (Shorter and Southworth, 2019). When working together, an Hsp70 and Hsp100 create a particularly potent aggregate disassembly machine referred to as a bi-chaperone disaggregase (Glover et al., 1998; Goloubinoff et al., 1999). Two of the best-studied examples of these chaperones are the *E. coli* Hsp70, known as DnaK, and the paired Hsp100 known as ClpB.

The Hsp70s and Hsp100s are both ATP-powered protein binding and restructuring machines (Mogk et al., 2018; Mayer and Gierasch, 2019; Shorter and Southworth, 2019). For Hsp70s like DnaK, ATP hydrolysis drives a functional cycle in which the chaperone alternates between tightly binding, then releasing, extended segments of substrate proteins that are enriched in hydrophobic amino acids (Flynn et al., 1989; Fourie et al., 1994; Gragerov and Gottesman, 1994; Schmid et al., 1994; Rudiger et al., 1997; Mayer et al., 2000). The targeted protein sequences are highly degenerate, occur at relatively high frequency in most proteins and are normally first bound by an Hsp40 (DnaJ in *E. coli*), prior to recruitment and transfer to the Hsp70 (Rudiger et al., 1997; Durme et al., 2009; Kampinga and Craig, 2010; Srinivasan et al., 2012). By contrast, the Hsp100s like ClpB are members of the AAA^+^ family of ATP-powered extrusion motors (Shorter and Southworth, 2019). These large homo-oligomeric, barrel-shaped proteins employ ATP hydrolysis to processively feed substrate proteins through their central pore as either linear chains or loops (Haslberger et al., 2008; Deville et al., 2017; Gates et al., 2017; Avellaneda et al., 2020). Importantly, initial substrate protein loading and activation of aggressive ATP turnover by ClpB requires, under most circumstances, direct binding between ClpB and DnaK (Acebrón et al., 2009; Oguchi et al., 2012; Winkler et al., 2012; Rosenzweig et al., 2013; Carroni et al., 2014). Current models suggest that DnaK binds first to exposed segments of an aggregated protein, followed by local recruitment of a ClpB oligomer. Subsequent transfer of the substrate protein to ClpB from DnaK is then coupled to activation of the feeding ATPase activity of the ClpB motor (Oguchi et al., 2012; Seyffer et al., 2012; Rosenzweig et al., 2013; Carroni et al., 2014).

Despite substantial progress in structural and functional characterization of both DnaK and ClpB, how they dismantle aggregates remains incompletely understood. This is, at least in part because protein aggregates and the complexes they form with molecular chaperones are typically complex and heterogeneous. Traditional bulk measurements like fluorescence or static light scattering can only capture the most limited average behavior of such systems. Depending on the underlying molecular distributions and associated dynamics, this ensemble average behavior can yield biased or even misleading interpretations (Kapanidis and Strick, 2009). The poorly understood manner in which a protein’s sequence specifies the types of aggregates that form under a given set of conditions is another serious confounding factor. In addition, many traditional disaggregation assays depend upon the recovery and detection of a native protein signature, typically enzymatic activity or a fluorescence response (Diamant et al., 2000; Zietkiewicz et al., 2006; Acebrón et al., 2009; Miot et al., 2011; Rosenzweig et al., 2013). While simple and sensitive, this approach fundamentally convolves two distinct events, disaggregation and folding, potentially resulting in loss of important mechanistic detail.

To circumvent these problems, we have applied a single particle technique known as Burst Analysis Spectroscopy (BAS) (Puchalla et al., 2008) to directly study protein disaggregation by the *E. coli* bi-chaperone disaggregase system. BAS provides a free solution, minimally perturbative approach to measuring the population-resolved kinetics of aggregate disassembly, as well as way to study the real-time binding stoichiometries of molecular chaperones in distinct aggregate sub-populations [multi-color BAS or MC-BAS (Shoup et al., 2021)]. To minimize the impact of protein sequence variability, we establish conditions that permit the controlled formation of two different aggregate types from the same aggregation-prone protein, ribulose-1,5-carboxylase oxygenase (RuBisCO) from *R. rubrum.* Using BAS, we demonstrate that the size of an aggregate nanoparticle appears to have only a modest impact on the susceptibly of the particle to disaggregation by the bi-chaperone system. Instead, internal structural properties of aggregates appear to be more important in determining how readily and how quickly an aggregate particle can be taken apart. We also show that this internal aggregate structure is not fixed, but changes on the minute time scale and in the complete absence of ongoing particle growth, altering the disassembly potential of aggregates even at the earliest stages of their formation.

## Results

### Non-Native RuBisCO Forms Distinctive Slow- and Fast-Growing Aggregates

RuBisCO from the nitrogen-fixing proteobacterium *R. rubrum* is a well-established model substrate protein for studying chaperonin-mediated protein folding (Goloubinoff et al., 1989a, 1989b; Rye et al., 1997; Brinker et al., 2001; Lin and Rye, 2004; Lin et al., 2008). We developed a set of conditions that yield two distinct types of protein aggregate from the same chemically denatured RuBisCO (Supplementary Figure S1). When acid-urea denatured RuBisCO is quickly diluted into standard buffer at 23°C, a large and rapid increase in static light scattering is observed (Supplementary Figure S1). This behavior is consistent with the rapid formation of aggregate particles with scattering cross-sections much larger than the native RuBisCO dimer. We refer to aggregates formed under these conditions as fast-growing or F-type aggregates. By contrast, when denatured RuBisCO is allowed to collapse into a kinetically trapped, late folding intermediate under conditions that initially inhibit aggregation (Lin and Rye, 2004), followed by rapid warming to 23°C, we observe a much slower and more modest increase in static light scattering (Supplementary Figure S1). We refer to aggregates formed under this second set of conditions as slow-growing or S-type aggregates.

**FIGURE 1:**
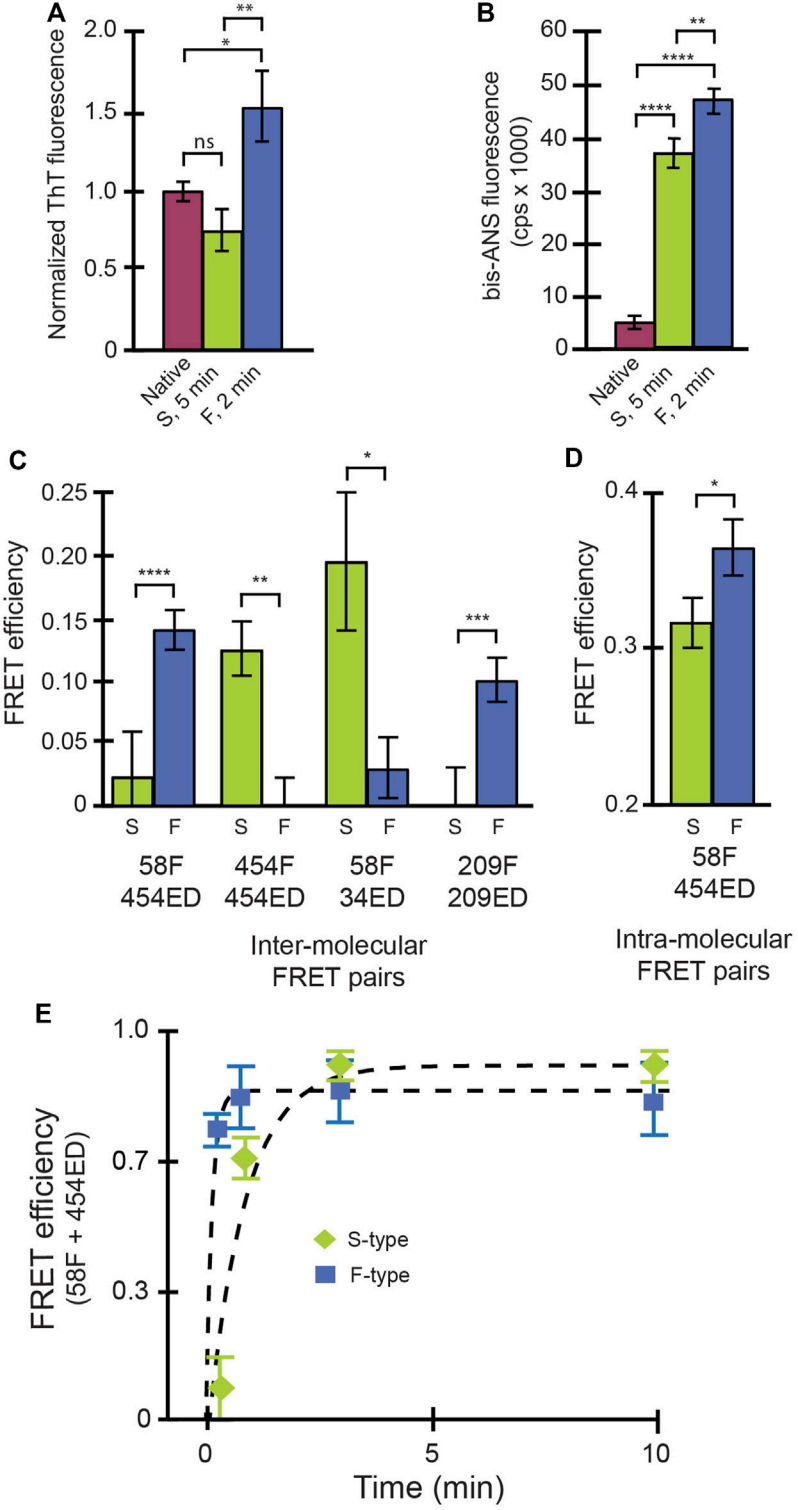
Conformational signatures of slow-growing (S-type) and fast-growing (F-type) RuBisCO aggregates in free solution. **(A)** Thioflavin T (ThT) binding to RuBisCO F-type aggregates (“F,” blue), S-type aggregates (“S,” green) and the native RuBisCO dimer (plum) is shown and provides an estimate of average beta sheet content (Sulatskaya et al., 2011). **(B)** Binding of bis-ANS, which provides an estimate of solvent accessible hydrophobic surface area (Rosen and Weber, 1969) of different RuBisCO aggregate types, is shown. **(C)** The average proximity of RuBisCO monomers within different aggregates was examined using a sensitized inter-molecular Förster resonance energy transfer (FRET). Different combinations of labeled monomers were employed, in which the amino acid position of the donor (*ED*) and acceptor (*F*) probes (indicated by numbers) was shifted. In order to increase the sensitivity of the assay to differences in relative dye position, 1:1 mixtures of donor- and acceptor-labeled monomers were mixed with unlabeled RuBisCO monomers (total labeled to unlabeled monomers of 1:4). **(D)** Average, relative proximity of the ends of the RuBisCO monomer was examined using intra-molecular FRET experiments, in which the donor and acceptor probes were site-specifically coupled to the same monomer (Lin and Rye, 2004; Lin et al., 2008). In each case, in order to minimize FRET between different monomers within the same aggregate particle, doubly labeled monomers were mixed with a large excess of unlabeled RuBisCO monomer (total labeled to unlabeled monomers of 1:9). For all experiments in **(A–D)**, F-type aggregates were examined at an aggregation time point of 2 min, while the S-type aggregates were examined at 5 min. For **(A,B)**, significance was assessed using a one-way ANOVA with *p*-values of * < 0.05, ** < 0.005, and **** < 0.0001. For **(C,D)**, significance was evaluated using a Student’s t-test, with *p*-values of * < 0.05, ** < 0.01, *** <0.005, and **** < 0.001. **(E)** The average initial growth rate of F-type (blue squares) and S-type aggregates (green diamonds) was examined using intermolecular FRET of a 1:1 mixture of 58F and 454ED labeled monomers with no unlabeled monomers added. In all cases, error bars show the standard deviation of *n* = 3 experimental replicates.

We next examined the conformational properties of both aggregate types using a series of free-solution assays. The F-type aggregates appear to be more enriched in β-sheet content, relative to either S-type aggregates or native RuBisCO, based on their thioflavin T (ThT) fluorescence enhancement (Sulatskaya et al., 2011) (Figure 1A). This observation suggests that the F-type RuBisCO aggregates might possess nascent β-amyloid character. Both S-type and F-type aggregates also show large fluorescence enhancements with bis-ANS, a small fluorochrome commonly employed to examine the extent of solvent exposed hydrophobic surface in protein structures (Rosen and Weber, 1969) (Figure 1B). Interestingly, the F-type aggregates display a modestly higher level of bis-ANS fluorescence compared with S-type aggregates. We also examined the average proximity of different segments of the RuBisCO chain, relative to other segments, using previously described inter- and intra-molecular ensemble FRET assays (Lin and Rye, 2004; Lin et al., 2008, 2013). For these experiments, small exogenous fluorescent probes were site-specifically coupled to engineered Cys residues at different locations throughout the RuBisCO sequence. Importantly, when every monomer in an aggregate is labeled, the observed FRET efficiencies tend to converge on smaller, average multi-donor/multi-acceptor distances. We therefore mixed labeled monomers with excess unlabeled monomers to increase the sensitivity of the assay to differences in average probe proximity in the aggregates. In every case, the S-type and F-type RuBisCO aggregates display substantial differences in sensitized FRET efficiency (Figures 1C,D), confirming that these aggregates are constructed from distinctive arrangements and, most likely, different conformations of the non-native RuBisCO monomers.

**FIGURE 2:**
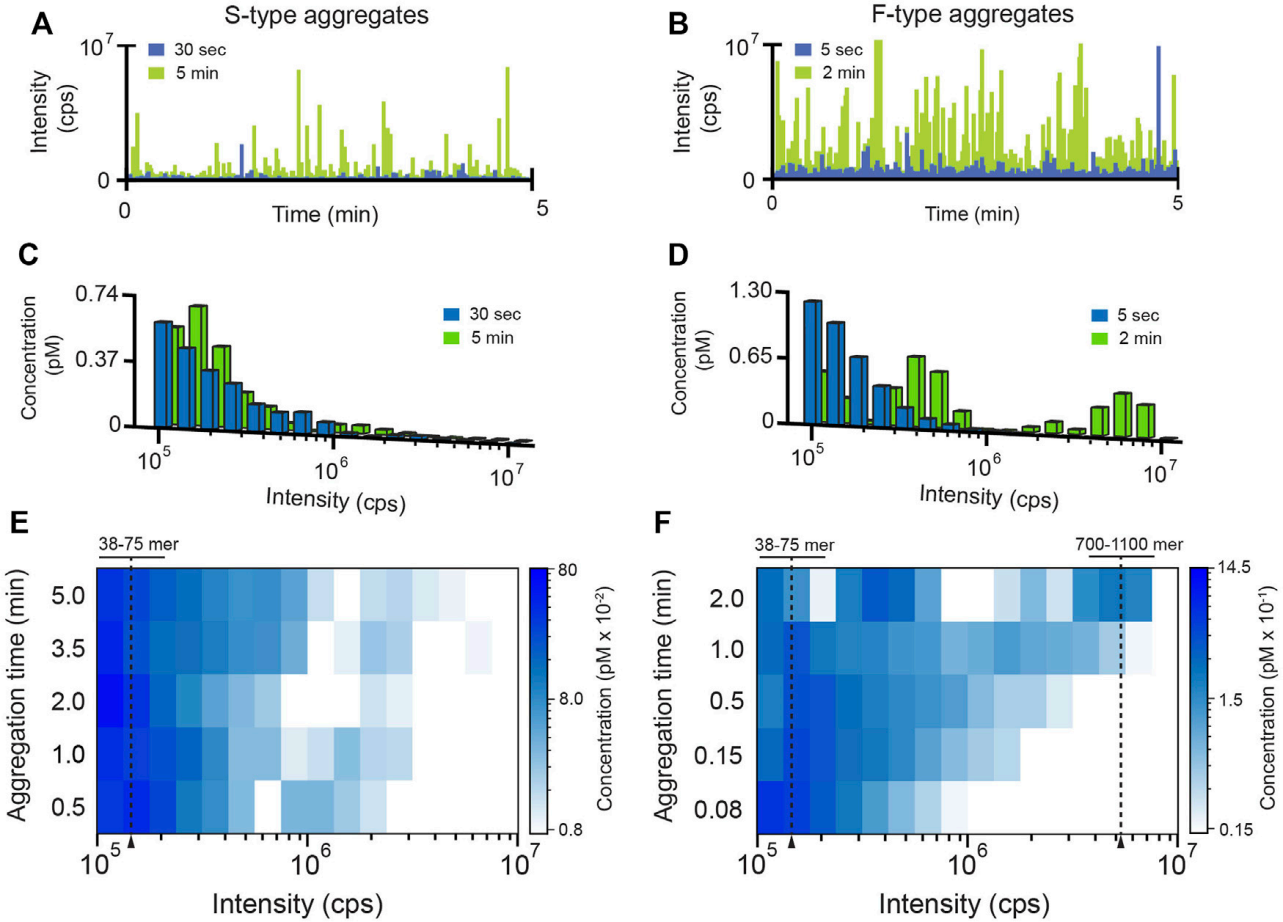
Characterization of RuBisCO aggregation pathways at the single-particle level. The population-resolved kinetics of aggregate growth (200 nM RuBisCO monomer) was examined by BAS (Puchalla et al., 2008). At each time point, aliquots of an aggregating, tetramethylrhodamine-labeled RuBisCO (RuBisCO-TMR) sample were removed and diluted 20-fold (10 nM final monomer) to halt aggregation and yield a total particle concentration appropriate for BAS. Raw photon histories were collected at early (blue) and late (green) aggregation time points for **(A)** S-type and **(B)** F-type aggregates. BAS was used to extract the underlying aggregate population distributions for the **(C)** S-type and **(D)** F-type aggregates. Time-dependent shifts in aggregate sub-populations were followed by sampling the aggregation reaction at higher time resolutions for **(E)** S-type and **(F)** F-type aggregates. For visualization purposes, high density BAS data is represented as a “heat map,” in which aggregate size is plotted on the *x*-axis, time or other variable is plotted on the *y*-axis, and the color of each bin is proportional to the concentration of particles at the given size. BAS histograms are a combination of *n* = 3 independent experimental replicates. Monomer number was established by calibrating the unitary brightness of the aggregate incorporated labeled monomer (see Supplementary Material; Supplementary Figure S2).

In order to gain additional insight into the behavior of the F- and S-type aggregates, we characterized their growth kinetics in greater detail. We first examined the average rate of aggregate formation using an inter-molecular FRET assay in which all RuBisCO monomers were labeled (Figure 1E). While the initial growth of both aggregate types is accompanied by a rapid increase in average FRET efficiency indicative of subunit contact and growth, the F-type aggregates form much more quickly than S-type aggregates, consistent with light scattering measurements (Supplementary Figure S1). However, because these ensemble FRET and light scattering signals cannot be easily assigned to specific aggregation steps or intermediates, we examined each aggregation pathway using BAS (Puchalla et al., 2008) (Figure 2). For these experiments, we employed a variant of RuBisCO carrying a single, covalently attached tetramethylrhodamine dye on each monomer (RuBisCO-TMR) (Lin and Rye, 2004; Lin et al., 2008, 2013). Initiation of either the F-type or S-type aggregation pathway results in the appearance of prominent fluorescent bursts that are much larger than those generated by the native RuBisCO-TMR dimer (Figures 2A,B; Supplementary Figure S2). Over time, the mean burst amplitude increases, indicative of aggregate growth as additional monomers are added to each particle (Figures 2A,B).

BAS permits the aggregate size and concentration distributions to be extracted from the observed fluorescent burst distributions (Figures 2C,D) (Puchalla et al., 2008). Importantly, the size distributions of the F-type and S-type aggregates display very different time-dependent behavior. While both aggregation pathways form particles that contain many RuBisCO monomers, the F-type aggregates grow much more rapidly (Figures 2E,F; Supplementary Figure S3), consistent with both ensemble FRET and light scattering measurements (Figure 1E; Supplementary Figure S1). However, while the FRET signal becomes insensitive to aggregate growth under these conditions between 0.5 and 2 min, BAS reveals that both aggregate types continue to grow. This observation is consistent with the rising light scattering response of the S-type and F-type aggregates at longer times, which is most simply interpreted as an increase in mean particle size (Supplementary Figure S1). Strikingly, while both aggregation pathways initially form particles that span a similar size range (mostly 38–75 monomers; see Supplementary Methods and Supplementary Figure S2), the F-type aggregates grow into larger structures very rapidly, while the S-type aggregates only slowly form larger particles (Figures 2C–F; Supplementary Figure S3). At the same time, while the S-type particle distribution slowly and continuously spreads toward moderately larger sizes, the F-type aggregates split into two sub-populations, one of which is only 3-5 fold larger than the starting population while the second grows into particles that are 10–30 fold larger (Figures 2C–F). To confirm that these BAS distributions reliably reflect aggregate particle mass distributions, we examined aggregating RuBisCO samples following centrifugation by BAS (Supplementary Figure S4). Increasing centrifugation velocities systematically deplete the right edge of the BAS intensity distributions, as expected for a sedimentation-induced loss of larger particles. Interestingly, while the smallest F-type particles fully sediment between 70 and 100,000 ×g, complete sedimentation of the smallest S-type particles requires centrifugation velocities over 3-fold higher.

### Aggregate Size Does Not Limit Disaggregation by KJEB

**FIGURE 3:**
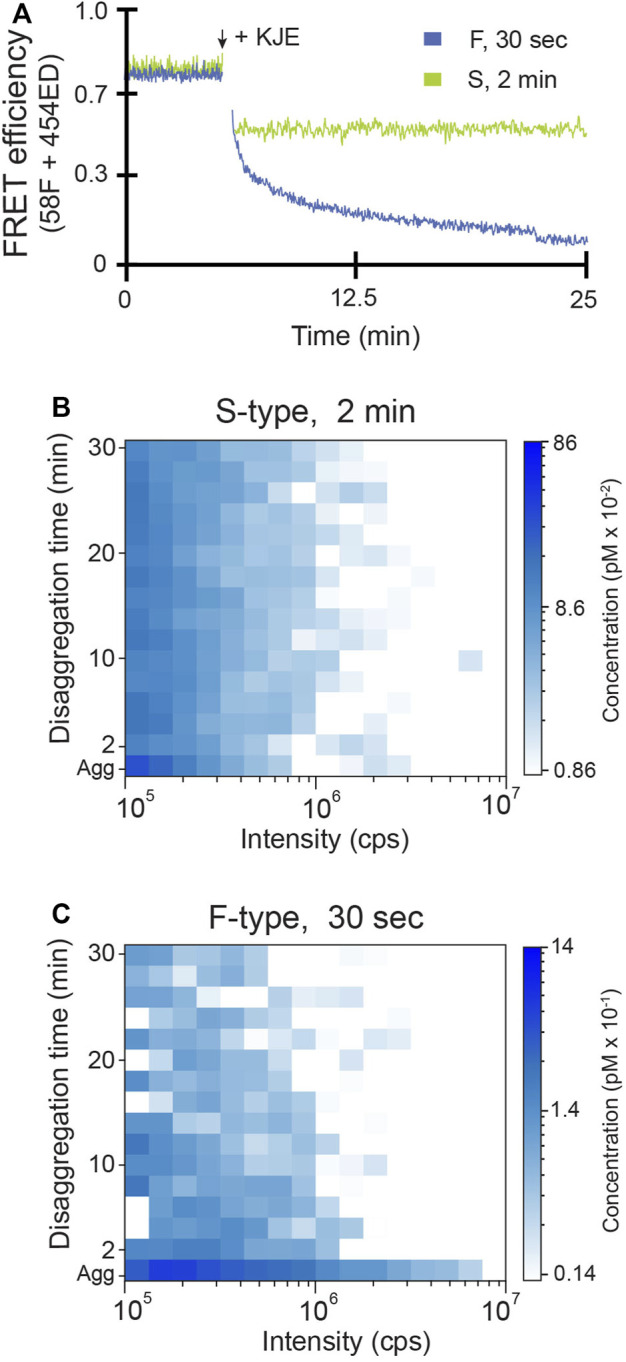
F-type RuBisCO aggregates can be partially disassembled by the KJE system alone. **(A)** The disassembly of early-stage S-type (green) and F-type (blue) aggregates by the DnaK system (KJE) was examined by inter-molecular FRET. Co-labeled aggregates (1:1 mixture of 58F and 454ED monomers) formed at early time points (2 min for S-type and 30 s for F-type) were mixed with 0.5 µM DnaK, 1 µM DnaJ and 1 µM GrpE plus 2 mM ATP and an ATP regeneration system. The population-resolved kinetics of RuBisCO-TMR aggregate disassembly was examined by BAS for **(B)** S-type and **(C)** F-type particles, under the same experimental conditions used for **(A)**. The aggregate particle distributions prior to the addition of KJE are shown (*Agg*). Following addition of KJE and ATP, aggregate disassembly was monitored in real time and BAS histograms were calculated for every 2 min of raw burst data. The histograms shown are a combination of n = 3 independent experimental replicates.

We next examined which aggregate properties, if any, determine how aggregate particles are dismantled by the disaggregase molecular chaperones. Using inter-molecular FRET, we first explored whether the bacterial Hsp70 system alone, consisting of DnaK, DnaJ and GrpE (KJE), can disassemble the F-type and S-type RuBisCO aggregates (Figure 3). We found that, in the presence of ATP, the addition of KJE to F-type aggregates results in a large, time-dependent decrease in FRET efficiency, consistent with extensive disaggregation (Figure 3A). However, S-type aggregates display only a small dead-time drop in transfer efficiency, with no subsequent change in signal (Figure 3A). The lack of a substantial decrease in FRET efficiency suggests that the S-type RuBisCO aggregates are substantially refractory to disaggregation by the KJE system alone.

Time-resolved BAS measurements confirm and extend these conclusions (Figures 3B,C). KJE-mediated disassembly of F-type RuBisCO aggregates is not confined to any particular particle size but appears to occur across the entire aggregate particle distribution, with the largest particles disappearing most rapidly (Figure 3C). With S-type aggregates, by contrast, BAS detects only a modest dead-time drop in the level of the smallest aggregates upon addition of the KJE system. The great majority of the S-type aggregate particles, over at least a 10-fold range of particle size, remain resistant to disassembly by KJE (Figure 3B).

**FIGURE 4:**
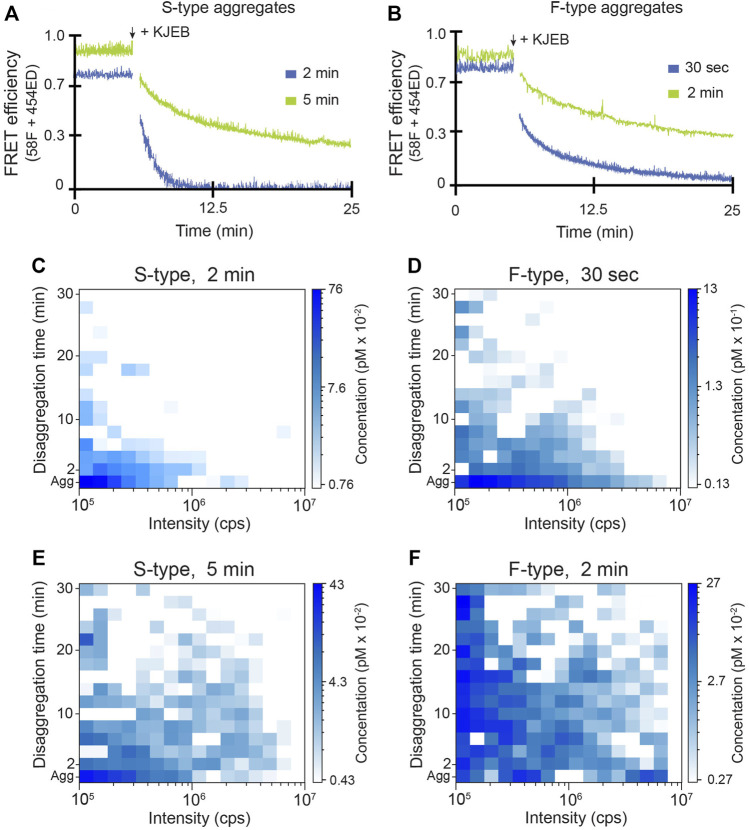
Disassembly of RuBisCO aggregates by the full KJEB bi-chaperone system. The disassembly of S-type **(A)** and F-type **(B)** aggregates by the full *E. coli* bi-chaperone system (KJEB) was monitored by inter-molecular FRET at both early (blue) and late (green) aggregation time points. Co-labeled aggregates (10 nM final total monomer) were created from a 1:1 mixture of 58F and 454ED monomers, followed by the addition of 0.5 µM DnaK, 1 µM DnaJ, 1 µM GrpE, and 300 nM ClpB plus 2 mM ATP and an ATP regeneration system. Disassembly was monitored in real time using a T-format spectrofluorometer. The population-resolved kinetics of RuBisCO-TMR aggregate disassembly for S-type and F-type aggregates by the KJEB system was examined by BAS at early **(C,D)** and late **(E,F)** aggregation time points. The aggregate particle distributions prior to the addition of KJE are shown (*Agg*). Following addition of KJEB and ATP, aggregate disassembly was monitored in real time, with BAS histograms calculated every 2 min of raw burst data. The histograms shown are a combination of *n* = 3 independent experimental replicates.

We next examined the impact of ClpB on disassembly of both S-type and F-type aggregates. As before, we first examined the average disaggregation response using inter-molecular FRET. While S-type aggregates are almost completely resistant to disassembly by the KJE system alone, addition of ClpB results in a large stimulation of the average disaggregation rate (Figure 4A). Examination of the same reaction by time-resolved BAS reveals the dramatic and rapid elimination of aggregate particles across the detectable size range (Figure 4C). By contrast, addition of ClpB to F-type aggregates in the presence of the KJE system results in a modest stimulation of the average disassembly rate, based on the change in FRET signal (Figure 4B). Direct observation of particle disassembly by BAS confirms this conclusion (Figure 4C). Importantly, in all cases, the disassembled RuBisCO monomers remain in solution as a concentrated sub-population of low brightness material that persists below the BAS detection threshold (Supplementary Figure S5). Detailed analysis of this product population suggests that the fully disaggregated RuBisCO exists mostly as monomers, likely in complex with one or more DnaK and/or DnaJ molecules (Supplementary Figure S5).

Because larger aggregate particles become more populated when aggregates are allowed to grow for longer times (Figure 2), we next examined the activity of the KJEB system at later aggregation time points. The average disassembly rate of both F-type and S-type aggregates, measured by FRET, slows when the aggregates are allowed to grow for longer times prior to the initiation of disaggregation (Figures 4A,B). Surprisingly, however, when similar disaggregation assays are conducted using time-resolved BAS, the entire range of aggregate particle sizes appears to be resistant to disassembly, not simply the larger particles (Figures 4E,F; Supplementary Figure S6). This result appears general for both the S-type and F-type aggregates, though the larger F-type aggregates appear to be somewhat more depleted, relative to the smaller F-type aggregate population, than are the larger S-type particles. Yet, even after 30 min of active disaggregation, substantial amounts of aggregate particles persist across the entire range of detectable sizes (Figures 4E,F).

### Aggregate Particles Do Not Re-Assemble During the Disaggregation Process

An important question is whether, and to what extent, protein aggregates can re-form while disaggregation is taking place. We addressed this question in two ways. First, we halted a disaggregation reaction at an intermediate time point by ATP depletion with hexokinase and glucose (Rye et al., 1997), measuring the aggregate particle distribution by BAS immediately before and after cessation of active chaperone turnover (Supplementary Figure S7). If a substantial fraction of the fully or partially disaggregated RuBisCO remained competent for re-aggregation, we would expect to see the aggregate particle distribution shift to larger sizes following the ATP quench. However, the aggregate particle distribution remains unchanged for up to 10 min (Supplementary Figure S7).

Second, we employed a two-color version of BAS (MC-BAS) (Shoup et al., 2021) to examine, in real time, the extent to which re-aggregation occurs during active disaggregation. For this experiment, two differently labeled RuBisCO samples were employed, one coupled to an Alexa488 dye (A488) and one coupled to an Alexa647 dye (A647), both at position 58. Because the spectral separation between these dyes is large, and they possess a very small Förster distance, the impact of FRET on the observed burst intensity distributions is negligible (Shoup et al., 2021). Importantly, prior BAS studies demonstrate that changing the chemical identity of the exogenous fluorescent probe at the C58 labeling site has little impact on the population-resolved growth kinetics of the RuBisCO aggregates (Shoup et al., 2021).

**FIGURE 5:**
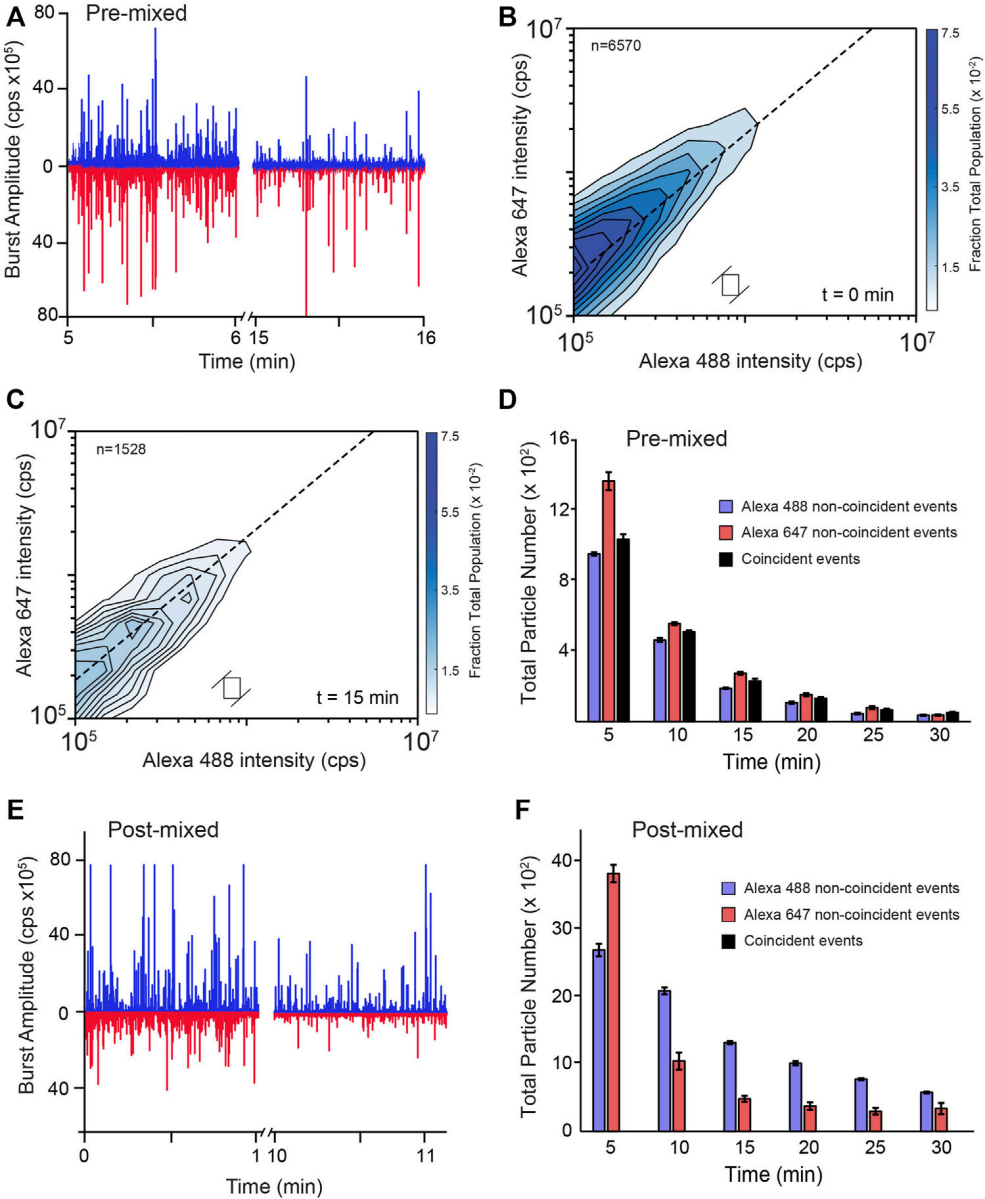
RuBisCO aggregates do not re-form during disaggregation by KJEB. **(A)** Raw photon histories from a multi-color BAS experiment (MC-BAS; Shoup et al., 2021) in which co-labeled, S-type RuBisCO aggregates were disassembled by KJEB. Aggregates were formed with a 1:1 mixture of two, differently labeled RuBisCO monomers, one carrying a single Alexa488 dye and the other a single Alexa647 dye (pre-mixed). The Alexa488 and Alexa647 dyes display little FRET coupling under these conditions (Shoup et al., 2021). Following formation of co-labeled aggregates (10 nM final total monomer), samples were supplemented with 1 µM DnaK, 2 µM DnaJ, 2 µM GrpE, and 200 nM ClpB, 2 mM ATP, and an ATP regeneration system. Aggregate disassembly was monitored in real time with MC-BAS. **(B)** The size, monomer stoichiometry, and concentration distributions of the co-labeled aggregate particles, prior to the initiation of disaggregation (*t* = 0), are displayed in a two-dimensional MC-BAS plot (Shoup et al., 2021). The dashed line shows the 1:1 monomer stoichiometry position. Positions along this diagonal are proportional to particle size and the spread of the distribution orthogonal to the dashed line is proportional to the relative stoichiometry of the two monomers. The total number of co-incident events in the data set is indicated (*n* = 6,570) and the size a raw MC-BAS pixel and its associated ratio range, which were used to calculate the interpolated contour plot, is illustrated with the open box. **(C)** The co-labeled MC-BAS distribution after 15 min of disassembly is shown. The MC-BAS histograms are a combination of *n* = 3 independent experimental replicates. **(D)** The number of co-labeled (i.e., coincident) aggregate particles observed as a function of time is shown, compared with the number of particles that are only detectable in one or the other channel (i.e., non-coincident). **(E)** Raw photon histories of an MC-BAS experiment in which each labeled monomer was separately aggregated and mixed with the KJEB system for 3 min, prior to co-mixing (post-mixed). Aggregation conditions, final total monomer and KJEB component concentrations are identical to the pre-mixed experiment **(A–D)**. **(F)** The number of particles detectable in only one or the other channel as a function of time is shown. No particles displaying significant coincidence were detectable over the course of the disassembly experiment. Error bars display the standard deviation for the total number of particles detected at each time point from *n* = 3 independent experimental replicates.

MC-BAS employs both the observed raw burst amplitudes and associated burst ratios to reconstruct the size and stoichiometry distributions of co-labeled particles (Shoup et al., 2021) When the RuBisCO-A488 and RuBisCO-A647 monomers are first co-aggregated, roughly a third of the total detected particles display strongly co-incident fluorescence bursts (Figures 5A–C). Following the addition of KJEB and ATP, the number of co-incident and non-coincident bursts decreases as the particles are disassembled (Figure 5D). However, when the differently labeled monomers are separately aggregated and exposed to KJEB for 3 min prior to co-mixing, no detectable co-incident particles are detected, even as the non-coincident aggregate particles are readily dismantled (Figures 5E,F). These observations demonstrate that no significant aggregate re-assembly takes place on the time scales of these disaggregation experiments.

### Internal Aggregate Structure is the Primary Constraint on Disassembly

**FIGURE 6:**
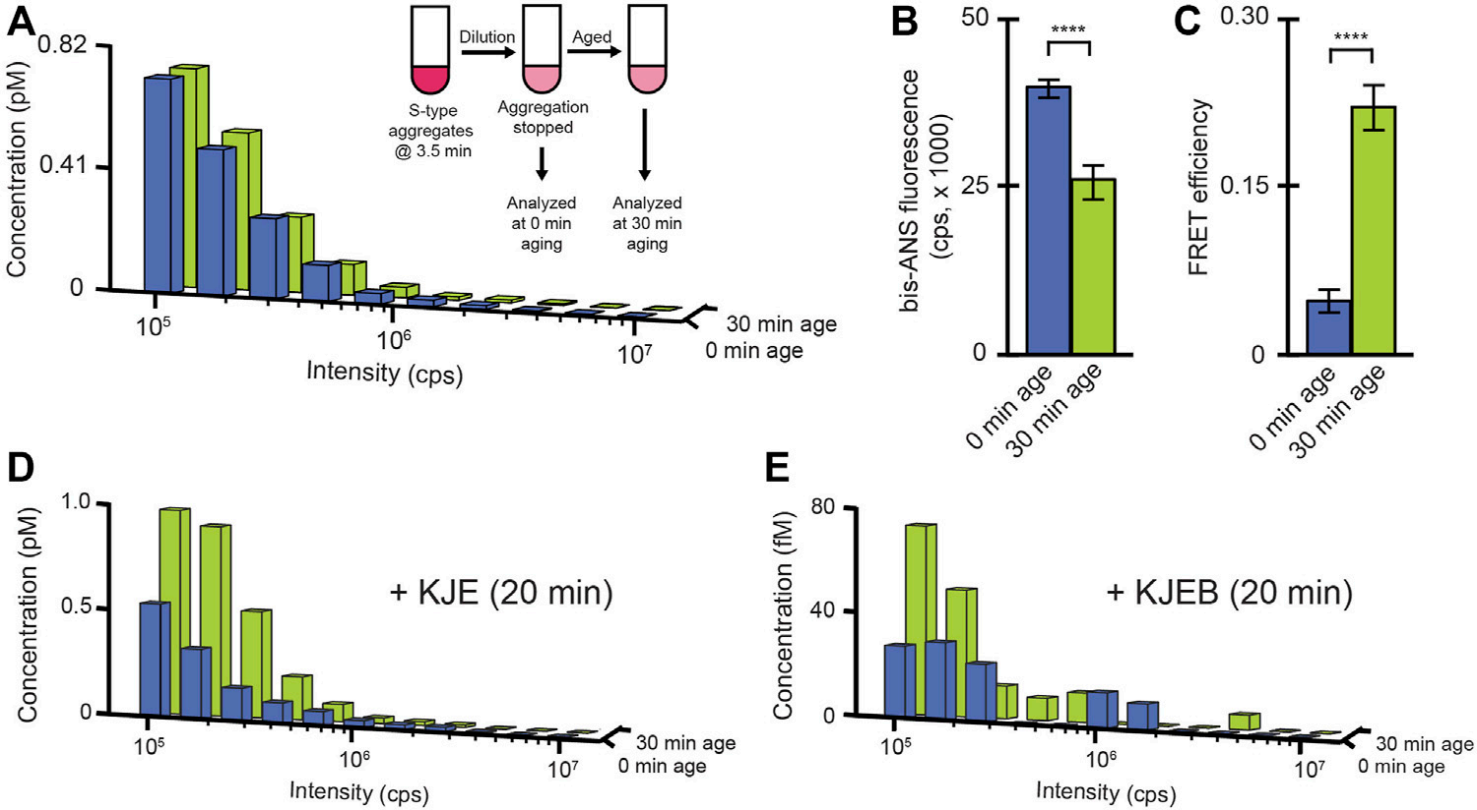
A conformational change in S-type aggregates inhibits disassembly. **(A)** S-type RuBisCO-TMR aggregates were grown for 3.5 min, then diluted to a final monomer concentration of 10 nM to halt growth (inset). Samples were then analyzed by BAS immediately (blue) or after a 30 min incubation at 23°C (green). Aging-induced changes in the average conformational properties of S-type aggregates were examined by bis-ANS binding **(B)** and sensitized inter-molecular FRET (c; 1:1 donor-to acceptor-labeled monomers at 1:4 total labeled to unlabeled monomers, 58F + 454ED). Error bars show the standard deviation of n = 3, independent experiments. Student’s t-test *p*-values of **** < 0.001. The impact of aging on disaggregation by the DnaK system alone **(D)** or the full bi-chaperone system **(E)** were examined by BAS following 20 min of exposure to the indicated chaperones and ATP. The BAS histograms are a combination of data from *n* = 3 independent experiments.

The time-dependent change in disaggregation susceptibility, for both S-type and F-type RuBisCO aggregates (Figure 4), suggest that whatever features limit aggregate particle disassembly, they are likely not fixed by the aggregation process alone. To examine this question, we developed an aggregate aging protocol. Early-stage S-type aggregates, which are initially susceptible to disaggregation, are rapidly diluted to halt their growth, followed by incubation at high dilution for an extended period (Figure 6A). We chose S-type aggregates for these experiments because their slower growth kinetics make technical execution of this experiment more straightforward. BAS measurements demonstrate that the observed aggregate particle size distributions before and after aging are identical (Figure 6A). At the same time, the aged S-type aggregates show clear signatures of altered structure. Specifically, aging results in both a decrease in bis-ANS fluorescence (Figure 6B) and increase in sensitized inter-molecular FRET between different monomers (Figure 6C). More significantly, however, aging causes a substantial reduction in the susceptibility of the aggregates to disassembly by either KJE alone or the full KJEB system (Figures 6D,E).

Slow or reduced disaggregation could, in principle, result from changes in the surface of aggregate particles that inhibit DnaK binding. We therefore employed a multi-color fluorescence burst coincidence experiment to examine DnaK binding to RuBisCO aggregates at different aging time points. We first created a fully functional, labeled version of DnaK *via* non-natural amino acid (azPhe) substitution at position 517, followed by site-specific, click-based coupling of the Alexa-like dye DBCO-488 (Wang et al., 2013). For the RuBisCO probe, we again employed the A647-labeled monomer to minimize FRET coupling.

**FIGURE 7:**
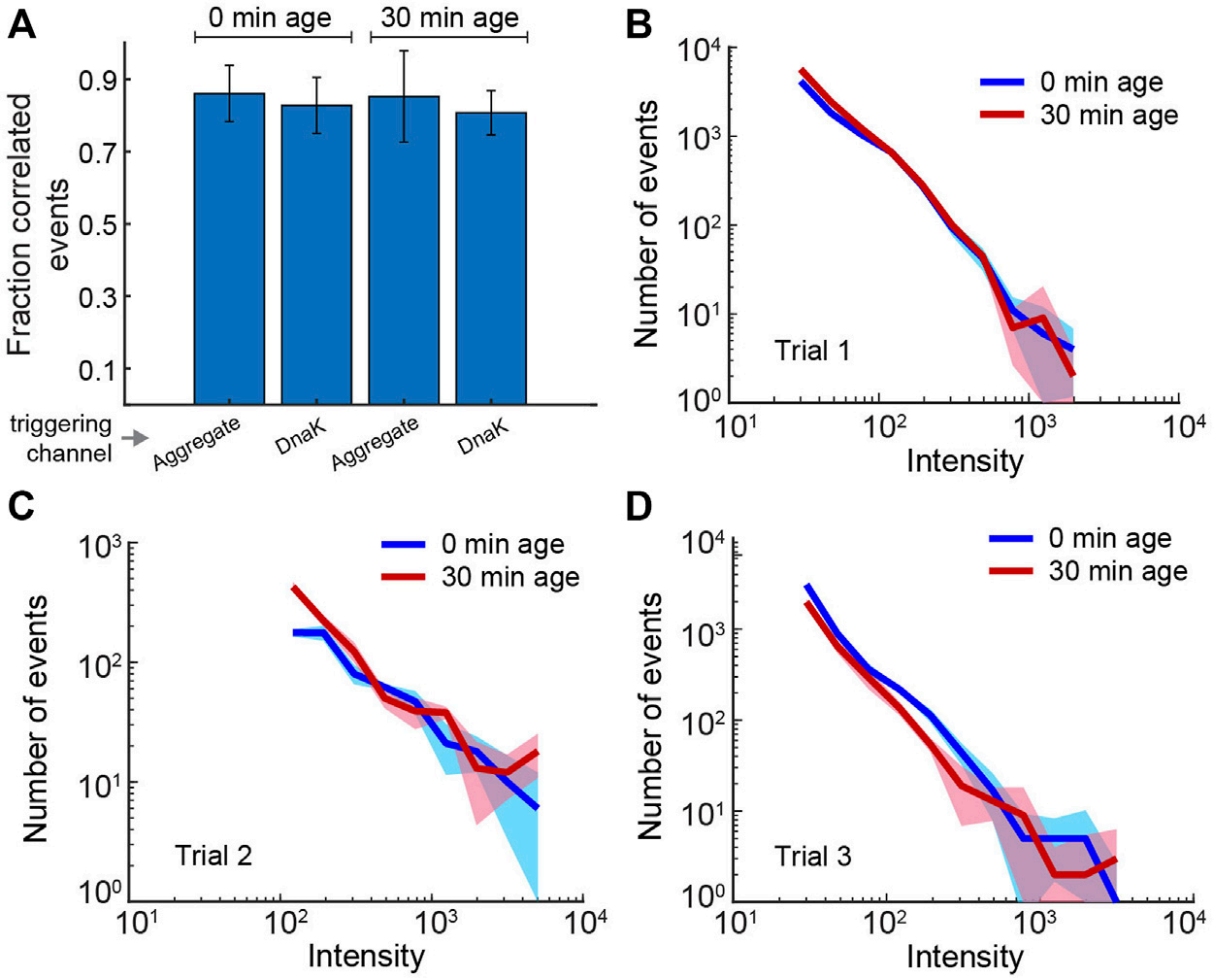
Binding of DnaK to S-type aggregates is not impacted by aggregate particle aging. Binding of fluorescent DnaK (DnaK517-DBCO488) to both aged and non-aged S-type aggregates (RuBisCO58-Alexa647) was examined using two-color burst co-incidence analysis. Aggregates were grown for 2 min, then diluted ×20 to stop further growth (10 nM final RuBisCO monomer). Samples were either immediately supplemented with the KJE system (10 nM DnaK517-DBCO488, 50 nM DnaJ, 50 nM GrpE and 2 mM ATP) or incubated at 23°C for 30 min prior to addition of the KJE system. Following an additional incubation at 23°C for 10 min, samples were treated with 0.05 U/µl hexokinase and 20 mM glucose to deplete ATP and stop chaperone turnover. **(A)** Each bar shows the fraction of detected events in the upper 50% of intensity that are coincident to within 0.5 ms. Because the efficiency of coincident binding was not known *a priori,* the average coincidence in each color channel (*aggregate, DnaK*) after 0 and 30 min of aging was separately considered. The reported uncertainties reflect the spread in coincidence for three experimental repeats and suggest no change in binding efficiency by DnaK upon aggregate aging. **(B–D)** The DnaK color channel burst intensity histograms (0.5 ms time bins; events greater than the minimum threshold—see *Methods*) for each of the three repeats used in panel **(A)**. The color bands represent statistical uncertainty based on the measured histogram bin variance. The overall stability of the measured distributions indicate that DnaK binding is not significantly impacted by aggregate aging. The high concentration of unbound DnaK in these experiment leads to background fluorescence that precludes extending to MC-BAS coincidence analysis.

Accumulation of DnaK-DBCO488 on aggregate particles could be observed as bursts in the DBCO488 channel that were co-incident with RuBisCO-Alexa647 bursts. However, we found that observation of robust burst co-incidence required addition of a significant excess of the labeled DnaK (5 nM final concentration). Because DnaK-DBCO488 possesses only a moderate specific brightness (Supplementary Figures S2E,F) and accumulated on aggregate particles to only modest levels under conditions that require a large unbound DnaK-DBCO488 pool, it was not possible to employ MC-BAS. We therefore evaluated the extent of DnaK binding examining 1) the fraction of total co-incident events and 2) the overall shape of the co-incident fluoresce burst histogram for non-aged and aged aggregates. Strikingly, the extent of DnaK binding to fresh and aged S-type aggregates appears to be essentially identical (Figure 7). Both in terms of the overall fraction of co-incident events and the burst amplitude distribution, aging appears to have no significant impact on the capacity of DnaK to recognize and interact with aggregates.

### The KJE System can Alter Aggregate Structure to Stimulate Disaggregation

**FIGURE 8:**
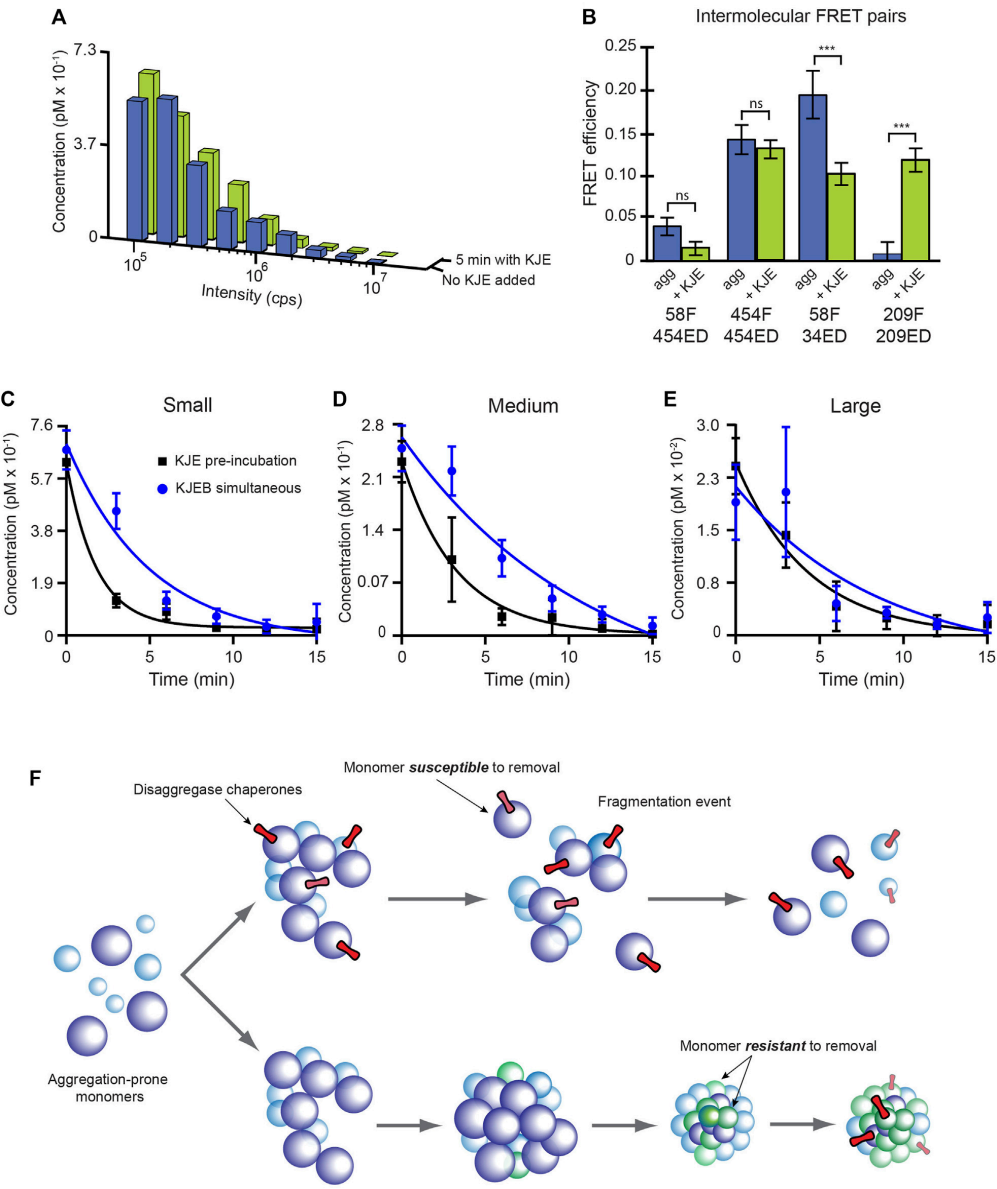
The KJE system enhances ClpB-mediated disaggregation by altering aggregate structure. **(A)** S-type RuBisCO-TMR aggregates grown for 3.5 min were incubated for 5 min either without (blue) or with (green) the KJE system alone (100 nM DnaK, 200 nM DnaJ, and 200 nM GrpE and 2 mM ATP). The aggregate size distribution observed for each sample, from a standard BAS measurement, is shown. **(B)** The average conformational properties of S-type aggregates were examined with sensitized intermolecular FRET after a 5 min incubation either with or without the KJE system. Co-labeled S-type aggregates (1:1 donor-to acceptor-labeled monomers at 1:4 total labeled to unlabeled monomers; 58F + 454ED) were grown for 3.5 min and then incubated for 5 min either with or without the KJE system alone, as described in panel **(A)**. Error bars show the standard deviation of *n* = 3 independent experiments and student’s t-test *p*-values of *** < 0.005. **(C,D)** Brief pre-incubation of S-type RuBisCO aggregates enhances the rate of ClpB-mediated disaggregation. S-type aggregates were grown for 3.5 min and supplemented with either the full bi-chaperone system in a single addition (KJEB simultaneous; blue) or the aggregates were pre-treated with the KJE system alone for 5 min, prior to the addition of ClpB (KJE pre-incubation, black). In each case, 100 nM DnaK, 200 nM DnaJ, 200 nM GrpE and 300 nM ClpB, along with 2 mM ATP and an ATP regeneration system was employed and aggregate disassembly was examined in real time by BAS. The observed aggregate size distributions were re-binned into coarse ranges of **(C)** small (43–135 mer), **(D)** medium (135–430 mer), and **(E)** large (430–1,400 mer) particles and their concentration plotted as a function of time. In each case, the observed disaggregation kinetics were fit to a single exponential rate law. Error bars show the standard deviation of *n* = 3 independent BAS disaggregation experiments. **(F)** Proposed model for the impact of internal aggregate structure on disaggregation by the KJEB bi-chaperone system. At early stages of RuBisCO aggregate growth (upper), the conformation of individual monomers, their contact surfaces with one another, and/or their overall organization permit maximally efficient disaggregation. The disruption forces applied by the KJE system either enhance or trap the global structure of the RuBisCO aggregate nanoparticle in a disassembly competent state that can be easily dismantled upon engagement of ClpB. Disassembly of large RuBisCO aggregate particles does not exclusively depend on monomer removal and can also proceed *via* cooperative release events and/or fragmentation. In the absence of early molecular chaperone action, the internal structure of the RuBisCO aggregate particles continues to evolve, even as the aggregate particles grow larger (lower). While this shift in internal structure does not dramatically alter surface features required for DnaK binding, it stabilizes the overall aggregate structure to such an extent that the full KJEB system can no longer dismantle the particle, irrespective of its size.

We next asked whether the disaggregase chaperones impact aggregate structure prior to the onset of disassembly, and if so, whether this is mechanistically consequential. Even though the KJE system alone only very weakly dismantles S-type particles, it appears to have significant broader impacts on other physical properties of the aggregates. Consistent with the observation that S-type aggregates are resistant to disassembly by KJE (Figure 3), a 5 min incubation of S-type aggregates with KJE and ATP has little impact on the aggregate particle distribution (Figure 8A). However, sensitized inter-molecular FRET assays, conducted under identical conditions, display clear signatures of altered aggregate conformation (Figure 8B). Of the four FRET pairs examined, two show substantial shifts in average site proximity following treatment with KJE and ATP. We also examined whether the KJE system is capable of structurally modifying the non-native RuBisCO monomer that forms S-type aggregates. Using four different intra-molecular FRET pairs, which collectively span the principal dimensions of the RuBisCO monomer, we first populated a kinetically trapped, non-native RuBisCO monomer at 4 °C (Lin and Rye, 2004; Lin et al., 2013). In every case, addition of KJE and ATP results in a decrease in the average proximity of the labeled regions of the RuBisCO monomer relative to one another, both at low temperature and at 25°C (Supplementary Figure S8). These results are consistent with prior observations of KJE-induced expansion of non-native rhodanese (Kellner et al., 2014) and non-native luciferase (Imamoglu et al., 2020) and strongly suggest that the KJE system can, in fact, directly alter the structure of a non-native RuBisCO monomer.

Pre-incubation of S-type aggregates with KJE and ATP results in a significant enhancement of disassembly upon subsequent addition of ClpB (Figures 8C–E). Slightly aged (5 min) S-type aggregates are readily dismantled when ClpB and the KJE system are added simultaneously, with disassembly half times between 3.5–8 min across all particle size ranges (Figures 8C–E). Strikingly, when the same aggregates are incubated with KJE and ATP for 5 min prior to the addition of ClpB, disassembly is accelerated by 2-4 fold, with the observed disassembly half times shrinking to 1–3 min across all particle sizes (Figures 8C–E).

## Discussion

Here we examined the disassembly of two types of protein aggregate by a bacterial bi-chaperone disaggregase at the single particle level. We employed *R. rubrum* RuBisCO as a model protein for these studies, because denatured forms of this enzyme can be shifted between two different aggregation pathways, permitting the creation of chemically identical, but physically distinct aggregate nanoparticles. We first classified these aggregates as either S-type or F-type based on their average growth rates and differential biophysical properties. Using BAS, we demonstrated that S-type and F-type aggregates follow divergent nanoparticles assembly pathways. Ensemble FRET and time-resolved BAS measurements further demonstrated that S-type and F-type aggregates possess different susceptibilities to disassembly by the bacterial disaggregase chaperones. Importantly, the observed disaggregation kinetics are not well predicted by particle size.

Aggregate particles that differ in monomer number by ∼20-fold, display disassembly times that differ by no more than 2 to 3-fold (Figures 4, 8; Supplementary Figure S6). This observation is surprising, given that particle size is generally assumed to be a key limiting constraint on aggregate disassembly (Diamant et al., 2000; Ben-Zvi et al., 2004; Zietkiewicz et al., 2006; Acebrón et al., 2009). For a simple disaggregation model where monomers are removed one at a time, the observed disaggregation time should roughly scale with the number of subunits if each monomer release event is independent of all others. Our observations violate this expectation and strongly suggest that disassembly of RuBisCO aggregates involves either 1) cooperative coupling between monomer release events and/or 2) particle fragmentation caused by disaggregase chaperone activity (Figure 8F). Indeed, we observed precisely this type of fragmentation behavior in our previous BAS studies of Hsc70-mediated clathrin disassembly (Krantz et al., 2013).

While particle size has little impact on disaggregation of either S-type or F-type aggregates, both aggregates display distinct disassembly kinetics and respond differently to the presence of ClpB (Figures 3, 4). Both aggregates also form particles that become resistant to disassembly over time (Figure 4). Thus, while their initiating conformational preferences do influence disassembly, these features do not fully predict disaggregation susceptibility. These observations suggest that important properties of an aggregate particle, which are essential for disassembly by the bi-chaperone system, are not fixed, but can change with time. Furthermore, these changes do not require ongoing particle growth (Figure 6), though they are likely to occur concurrently (Figure 4; Supplementary Figure S6) and could well influence one another. Indeed, in a prior study of aggregate particle growth using MC-BAS, we detected signatures of biased aggregate growth by RuBisCO F-type aggregates linked to aggregate maturation time (Shoup et al., 2021). In that study, low-order aggregate particles, which had grown and matured for only 15–20 s, preferentially coalesced with one another rather than incorporate fresh, non-native monomers.

Overall, these observations can be explained if both the F-type and S-type aggregates possess a set of hidden, but critical, structural features that are necessary for disassembly, but which can also change rapidly over time. The differences we observe in ThT binding, bis-ANS binding and sensitized intra- and inter-molecular FRET are consistent with this idea (Figures 1, 6). Two models could then explain how structural changes in these aggregate particles could inhibit disassembly by the KJEB system. First, changes in the external surface structure of the aggregate could prevent binding of the essential, initiating DnaK or DnaJ molecular chaperones and thus block disaggregation. However, our observations that aggregate aging, which impacts both aggregate disassembly and structure but does not alter DnaK binding (Figures 6, 7), are not consistent with this model. Alternately, changes in internal aggregate structure might so strengthen the interactions holding the particle together that the KJEB system can no longer supply sufficient force to disrupt them. Our observations, particularly with S-type aggregates, are most consistent with this second, internal structure maturation model (Figure 8F).

How the disaggregase chaperones target and modify these critical structural features remains incompletely understood. While the presence of the bacterial Hsp70 system (KJE) is essential in all cases, aggressive disassembly of S-type RuBisCO aggregates requires the presence ClpB (Figures 3, 4). At the same time, disaggregation of F-type aggregates is slower, but still substantial, in the absence of this Hsp100 machine (Figure 3). These observations are consistent with prior studies showing that the KJE system alone possesses potent intrinsic disaggregase activity against certain types of aggregates, under certain conditions, while obligately requiring the ClpB system to dismantle other aggregates under other conditions (Mogk et al., 1999; Diamant et al., 2000; Ben-Zvi et al., 2004; Weibezahn et al., 2004; Zietkiewicz et al., 2004, 2006; Acebrón et al., 2009; Sielaff and Tsai, 2010; Miot et al., 2011; Winkler et al., 2012). Why some protein aggregates are quickly dismantled, while others are almost completely refractory to disaggregation remains unresolved. Though our RuBisCO-based observations cannot be proven to generalize to other aggregation-prone proteins without additional studies, they suggest that differences in the internal structure of an aggregate particle can, at least in principle, play a critical limiting role in chaperone-mediated disaggregation.

Importantly, disaggregation generally requires that the KJE system interact first with an aggregate, prior to recruitment of ClpB (Acebrón et al., 2009; Winkler et al., 2012). Direct physical contact between Hsp70s like DnaK and their cognate Hsp100s, which is linked to substrate protein transfer between the chaperones and activation of the Hsp100 ATP turnover and threading activity, is likely the basis for this binding order (Oguchi et al., 2012; Seyffer et al., 2012; Rosenzweig et al., 2013; Carroni et al., 2014). Our observations suggest a potential second role for initial aggregate engagement by KJE, one linked to this system’s intrinsic disaggregase activity. Partial disruption of an aggregate’s global structure upon DnaK binding could be important for preparing or “loosening” internal aggregate structure prior to ClpB engagement. The accelerated disassembly we observe when S-type aggregates are pre-incubated with KJE prior to addition of ClpB, along with attendant, pre-disassembly changes in aggregate structure (Figure 6) and the inherent capacity of the KJE system to restructure the non-native RuBisCO monomer (Supplementary Figure S8), are consistent with this idea.

Interestingly, the stimulatory effect of KJE pre-incubation appears strongest for small aggregate particles and dissipates as the particles become larger (Figures 8C–E). This observation suggests a possible model for physical coupling between the monomers of the S-type aggregates. The aggregate surface layer, where the KJE chaperone system is engaged, is likely to be most strongly coupled to the next closest, inner layer(s) of monomers that make direct physical contact with the surface monomers. Application of a disruptive force at the surface by the KJE system could then propagate inward to the next set of monomers, just below the layer being directly attacked by the disaggregase chaperones. Because the S-type RuBisCO aggregates grow from collapsed, globular folding intermediates, they can be reasonably approximated as spherical. A comparison of the small aggregate populations observed here (50–75-mers) to the classic sphere packing problem (Conway and Sloane, 1999; Brass, et al., 2005) then provides potential insight into the appearance of internal layers. With optimal packing, the first two layered aggregate structures would correspond to 13 and 64-mers, respectively, suggesting the mitigated stimulatory response of DnaK could well be driven by the transition to additional buried structure. Time-dependent changes in the strength of these inter-layer couplings could also underpin the inhibitory effects of aggregate aging. Such a model would predict that the general structural disruption action that the KJE system can apply should dissipate as both a function of aggregate particle size and time.

Whether DnaK-induced structural disruption is required for cooperative monomer release or particle fragmentation, and the extent to which ClpB is required for full particle disassembly, are unclear and will require further investigation. However, at least for RuBisCO, productive protein folding is not required for efficient disaggregation. RuBisCO is an obligate Hsp60 substrate protein and cannot achieve its native state under these conditions without the assistance of a chaperonin, such as the bacterial GroELS system (Goloubinoff et al., 1989a, 1989b; Rye et al., 1997). Even so, the disassembled RuBisCO monomers do not re-aggregate (Figure 5; Supplementary Figure S7) on the time scale of these assays and appear to remain in solution, mostly likely as chaperone bound monomers (Supplementary Figure S5).

Significantly, metazoans possess no Hsp100 homolog known to participate in cytoplasmic protein disaggregation (Nillegoda et al., 2018). Instead, these organisms employ highly effective disaggregase systems constructed from an elaborated set of Hsp40 proteins and a modified Hsp70 (an Hsp110) that acts as a nucleotide exchange factor for the core Hsp70 (Nillegoda et al., 2015; Faust et al., 2020). How aggregate binding by metazoan Hsp70s is so efficiently rectified into disaggregase activity, in the absence of an Hsp100 pulling motor, is not fully understood, though entropic pulling forces are suspected to be key (Rios et al., 2006; Rios and Barducci, 2014; Sousa et al., 2016). Our observations that the simpler bacterial KJE system appears to induce broad alterations in RuBisCO aggregate structure, which can be coupled to enhanced disassembly, is suggestive of a general aggregate disruption mechanism by Hsp70s, one that has been dramatically elaborated in metazoans (Nillegoda et al., 2015; Faust et al., 2020). Understanding the detailed nature of this effect, whether it is generally applicable to other protein aggregates and disaggregase systems, and how it is coupled to efficient aggregate disassembly are key questions for future investigation.

## Experimental Methods

### Protein Expression and Purification

Wild-type *R. rubrum* RuBisCO (58C) and various mutants (454C, 209C, 356C, 58A/454C, 58A/34C, 58A/209C, and 58A/454C/34C) were expressed and purified as previously described (Lin and Rye, 2004; Lin et al., 2008, 2013). The gene for DnaK was amplified from *E. coli* genomic DNA and sub-cloned into the pPROEX HTb vector for expression in *E. coli* BL21. The genes for ClpB, DnaJ, and GrpE, sub-cloned into a pET 151/D-TOPO vector for expression in BL21DE3, were generous gifts from Dr. Steve Burston (University of Bristol, United Kingdom). Expression and purification of DnaK, ClpB, GrpE, and DnaJ was carried out similarly to a previously described procedure (Sweeny et al., 2011), with some modifications (see Supplementary Material).

A fluorescent variant of DnaK was created using non-natural amino acid substitution and click chemistry. DnaK carrying a p-azido-phenylalanine (azPhe) substitution at position 517 was generated by using a previously described approach (Wang et al., 2011, 2013), with slight modification. In brief, the arginine codon at position 517 of DnaK was mutated to an amber codon (UAG), yielding a DnaKam-pProex-HTB expression plasmid. *E. coli* BL21 (DE3) was transformed with both DnaKam-pProex-HTB and a second plasmid (AzPheRS/mutRNACUA-pYC-J17) carrying both a mutant tyrosyl-tRNA synthetase and a mutant tyrosine amber suppressor tRNA. IPTG induction in the presence of p-azido-L-phenylalanine allowed suppression of the amber codon and incorporation of azPhe at position 517. Purification of DnaK517-azPhe was essentially identical to wild type DnaK, except that the concentration of beta-mercaptoethanol in all buffers was lowered to 0.5 mM to reduce thiol-induced inactivation of the aryl azide moiety.

### Protein Labeling

The thiol reactive dyes fluorescein-5-maleimide (F), IAEDANS (ED), tetramethylrhodamine-5-maleimide (TMR), AlexaFluor-488-maleimide (Alexa488), and AlexaFluor-647-maleimide (Alexa647) were obtained as dry powders from ThermoFisher/Molecular Probes. Labeling, purification and characterization of both single-labeled and double-labeled fluorescent RuBisCO variants was carried out as previously described (Rye, 2001; Lin and Rye, 2004; Lin et al., 2008, 2013; Weaver and Rye, 2014; Shoup et al., 2021).

AZDye 488-DBCO was obtained as a dry powered from Click Chemistry Tools. Fluorescent DnaK was created by reacting the non-natural aryl azide moiety of DnaK517-azPhe with the strained alkyne group of the AzDye 488-DBCO (Wang et al., 2013). In brief, the 488-DBCO dye, reconstituted in DMSO, was mixed with 100 µM DnaK517-azPhe in 50 mM Tris, pH 7.4, 100 mM KCl, 0.5 mM EDTA in two, sequential additions of 100 µM each, with a 2 h incubation at 23°C after each addition (total final molar excess of AzDye 488-DBCO to DnaK517-azPhe of 2:1). Contaminating, truncated DnaK from failed suppression of the amber stop codon was separated from labeled DnaK517-488-DBCO with a Superose 6 gel filtration column (Cytiva) in 50 mM HEPES, pH 7.6, 150 mM KOAc, 10 mM Mg(OAC)_2_, 5 mM ATP, and 2 mM DTT. Inclusion of ATP in the column running buffer inhibited binding of the truncated DnaK contaminant to DnaK517-488-DBCO. DnaK517-488-DBCO displayed steady state ATPase kinetics (both intrinsic and stimulated) that were identical to wild type DnaK. In addition, DnaK517-488-DBCO supported enhanced folding of denatured luciferase and displayed RuBisCO disaggregation kinetics that were identical to wild type DnaK.

### RuBisCO Aggregation

Naive RuBisCO was denatured by dilution into acid-urea buffer (25 mM glycine-phosphate, pH 2.0, 8 M urea) to a final concentration of 10 μM, followed by incubation for 30 min at 23°C. F-type aggregation was initiated by very rapid dilution of denatured RuBisCO (200 nM final monomer) into sample buffer (50 mM HEPES, pH 7.6, 150 mM KOAc, 10 mM Mg(OAc)_2_, and 2 mM DTT) at 23°C. Growth was halted at different aggregation time points by a further 20-fold dilution into sample buffer to yield a final RuBisCO monomer concentration of 10 nM. S-type aggregation was initiated by first diluting denatured RuBisCO monomer (200 nM final monomer) into sample buffer at 4°C to populate a kinetically trapped, non-native RuBisCO monomer resistant to aggregation at low temperature (Lin and Rye, 2004). Following a 2 min incubation on ice, the sample was rapidly warmed to 23°C to trigger aggregation, which was halted at different time points by a further 20-fold dilution into sample buffer at 23°C.

### Thioflavin T Binding Assay

Thioflavin T was obtained as a dry powder from Sigma-Aldrich. A working stock (10 mM) was prepared in sample buffer (50 mM HEPES, pH 7.6, 150 mM KOAc, 10 mM Mg(OAC)_2_, and 2 mM DTT). For experiments with native RuBisCO, ThT (1 µM final) was mixed with the native RuBisCO dimer (100 nM final monomer) in sample buffer. S-type aggregates were grown for 5 min and F-type aggregates for 2 min at 200 nM monomer in each case. Aggregate samples were then mixed (1:1) with ThT (2 µM). In all cases, samples were incubated with ThT for 30 s at 23°C before the fluorescence emission spectra were recorded using a thermally jacketed sample cuvette at 23°C in a steady state, photon-counting fluorometer (HORIBA/Photon Technology International). Excitation was set for 450 nm and emission was integrated from 480–490 nm and corrected for the emission of ThT alone. All experiments were repeated a minimum of 3 times.

### Bis-ANS Binding Assay

4,4′-Dianilino-1,1′-Binaphthyl-5,5′-Disulfonic Acid (bis-ANS) was obtained as a dry powder from ThermoFisher and was prepared in sample buffer as a 200 µM working stock. For experiments with native RuBisCO, 5 µM bis-ANS was mixed with 100 nM native RuBisCO (monomer) in sample buffer. RuBisCO aggregates were prepared using the same 1:1 protocol outlined above for ThT experiments, except that 10 µM bis-ANS in sample buffer was used for the final dilution buffer (5 µM bis-ANS final). In all cases, samples were incubated for 1 min at 23°C and the fluorescence emission spectra recorded using a thermally jacketed sample cuvette at 23°C. Excitation was set at 375 nm and the emission was integrated from 470–540 nm and corrected for the emission of bis-ANS alone. All experiments were repeated a minimum of 3 times.

### FRET-Based Aggregation and Disaggregation Assays

Samples of denatured donor-only (ED), acceptor-only (F), double-labeled (donor and acceptor) and unlabeled RuBisCO monomers were prepared by dilution of native dimers into acid-urea buffer as outlined above. All FRET experiments employed matched sets of unlabeled, donor-only, acceptor-only and donor-acceptor samples, where the final RuBisCO monomer concentration was the same in each case. For donor-only and acceptor-only samples, unlabeled RuBisCO was used to equalize the final total monomer concentration across each sample set. For experiments employing inter-molecular FRET to follow aggregation or disaggregation kinetics, all monomers carried a fluorescent probe, with the donor- and acceptor-labeled samples mixed at 1:1. For both inter- and intra-molecular FRET experiments where structural differences in aggregates were examined using FRET, unlabeled RuBisCO was used as a diluent to reduce the average number of labeled monomers per aggregate particle while maintaining the total monomer concentration at the level needed for repeatable aggregate growth. In these cases, the fraction of labeled monomers was reduced to 10% of the total RuBisCO protein. Mixtures of labeled and/or unlabeled RuBisCO monomers at final desired ratios were prepared in acid-urea at a final total monomer concentration of 10 µM. Growth of S-type or F-type aggregates from these mixed samples was then triggered by dilution into sample buffer and halted by further dilution as described above.

Donor-side transfer efficiencies for different FRET pairs, in different aggregate types, were derived from the fluorescence emission spectra of donor-only and donor-acceptor samples using a thermally jacketed sample cuvette at 23°C. Excitation was set at 336 nm and the emission was integrated from 430–450 nm. FRET efficiencies were calculated from the background corrected, integrated donor emission spectra of donor-only and donor-plus-acceptor samples (Lin and Rye, 2004; Lakowicz, 2006). Sample background was determined from the emission spectra of a matched aggregate sample that contained only unlabeled RuBisCO monomers. The observed emission of acceptor-only samples in the acceptor emission band, following illumination at the donor excitation wavelength, was employed as an internal reference to check energy transfer *via* enhanced acceptor emission in donor-acceptor samples.

For measurement of disaggregation kinetics by FRET, aggregated samples were first grown from matched donor-only and donor-acceptor samples as described above. Aggregates were then rapidly mixed with a reaction cocktail containing the disaggregase chaperones and ATP in a thermally jacketed fluorometer cuvette. An ATP regeneration system, consisting of 5 U/ml of creatine kinase and 3 µM creatine phosphate, was also added to every sample to maintain a constant level of ATP during the measurements. The donor-side fluorescence emission of each sample was monitored as a function of time, with excitation set at 336 nm and emission was integrated from 430–450 nm. All FRET experiments were repeated a minimum of 3 times.

### BAS and MC-BAS

Fluorescence burst measurements were taken with a custom-built, multi-channel BAS microscope Puchalla et al., 2008; Shoup et al., 2021). RuBisCO aggregates were prepared either from Rub58-TMR, Rub58-Alexa488 or Rub58-Alexa647 monomers. For single color BAS aggregation experiments with Rub58-TMR (e.g., Figure 2), aggregate growth was initiated in a master sample at 23°C as described above. At desired aggregation time points, an aliquot was removed, and aggregation was halted by dilution (10 nM final monomer). A 10 µl aliquot of the diluted mixture was placed on a highly cleaned, BSA-blocked coverslip mounted on the microscope stage, which was then covered with a humidity chamber. Fluorescence burst data was recorded using a linear sample flow rate of 500 μm/s and 50 µW (at the sample) from a 561 nm diode-pumped solid-state laser. The raw photon history was recorded for a minimum of 3–5 min for each sample and the observed particle population distribution then extracted with BAS (Puchalla et al., 2008).

BAS disaggregation experiments using Rub58-TMR were conducted by diluting the labeled aggregates (10 nM final monomer) into a reaction cocktail containing disaggregase chaperones, ATP and an ATP regeneration system (5 U/ml creatine kinase and 3 µM creatine phosphate). The sample was immediately loaded on a blocked coverslip and burst data was recorded continuously over 20–30 min. Each data record was then segmented into 2–3 min blocks so that each coarse binned time point contained a sufficient number of burst events for robust analysis. For multi-color BAS (MC-BAS) disaggregation experiments, denatured samples of Rub58-Alexa488 or Rub58-Alexa647 were prepared as outlined above. For co-aggregation experiments, a 1:1 mixture of the denatured, differently monomers was prepared in acid-urea, which was then diluted into cold sample buffer to populate the kinetically trapped RuBisCO monomer. This sample was warmed to induce S-type aggregate formation and at the desired aggregation time, aliquots of this mixture were removed and diluted (10 nM final monomer concentration) into buffer containing the disaggregase chaperones and ATP. Mixtures of pre-formed, differentially labeled RuBisCO aggregates were created by first following the S-type aggregate preparation protocol for Rub58-Alexa488 and Rub58-Alexa647 samples separately. Following 5 min of aggregate growth at 23°C, the samples were mixed (1:1) and an aliquot of the mixed sample was then diluted (10 nM final monomer concentration) into buffer containing the disaggregase chaperones and ATP. For both types of experiment, samples were loaded onto a mounted and blocked coverslip immediately following chaperone addition. Burst data was collected continuously for 20–30 min with excitation from co-aligned 488 nm and 642 nm lasers (50 µW each) and each data record was segmented into 2–3 min coarse bins and processed using MC-BAS as previously described (Shoup et al., 2021). All BAS experiment were repeated a minimum of 3 times and all population distributions shown illustrate the mean behavior of these combined experimental replicates.

### Two-Color Burst Co-Incidence Analysis of DnaK Binding to Aggregates

Binding of fluorescent DnaK (DnaK517-DBCO488) to fluorescent RuBisCO aggregates labeled with Alexa647 at position 58 was examined using the same multi-channel microscope employed for MC-BAS. S-type RuBisCO aggregates were first grown for 2 min at a RuBisCO monomer concentration of 200 nM and were then diluted ×20 to halt aggregate growth (10 nM final RuBisCO monomer). Aggregates were either immediately supplemented with the KJE system (10 nM DnaK517-DBCO488, 50 nM DnaJ, 50 nM GrpE and 2 mM ATP) or were incubated at 23°C for 30 min prior to addition of the KJE system. Samples were then incubated for and additional 10 min at 23°C followed by treatment with 0.05 U/µl hexokinase and 20 mM glucose in order to deplete ATP and prevent further chaperone turnover. Each aggregate/chaperone mixture was then loaded on blocked coverslip and data was collected using a standard BAS format.

For each collected time stream, a background count value was determined from the mean of the data time stream, where time bins with values greater than five times the global rms value are masked. An upper bound value was set as the median count value of the largest thirty bin counts. A minimum threshold for significant events was then set at twice the mean of the masked time stream or 1% of the upper bound, whichever was larger. Bins with the same time registration in the two-color channels were considered coincident events if each contained photon counts greater than 50% of the upper bound for that channel. The minimum threshold was always less than 50% of the upper bound. The fraction of coincident bins for a given color channel was then the number of coincident bins measured in the experiment divided by the number of events in that color channel greater than 50% of the upper bound for that channel. The reported event histograms in Figure 7 are created from all bins above the minimum threshold (coincident or not) and, in general, do not represent single-particle events.

## Data Availability

The original contributions presented in the study are included in the article/Supplementary Material, further inquiries can be directed to the corresponding author.
