# Supplementary material for: The Impact of Hidden Structure on Aggregate Disassembly by Molecular Chaperones
Source: Front Mol Biosci. 2022 Jul 7;9:915307. doi: 10.3389/fmolb.2022.915307 (PMC9302491; doi:10.3389/fmolb.2022.915307)
Supplement: Supplementary file 1 [file DataSheet1.docx]

**SUPPLEMENTAL INFORMATION**


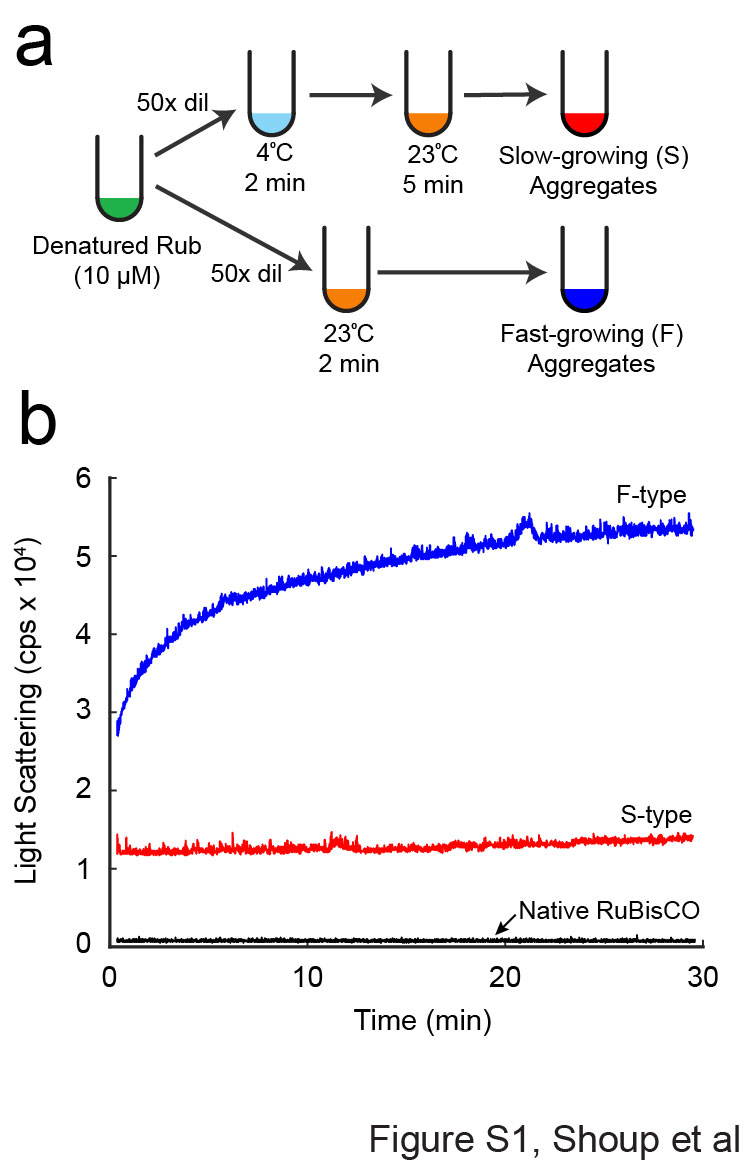


**Supplemental Figure 1. RuBisCO can form both S-type** **and F-type** **aggregates.** (a) Schematic illustrating the mixing protocols used to prepare protein aggregates from *R. rubrum* RuBisCO. Direct dilution from denaturant into buffer at 23 °C results in formation of fast-growing (‘F-type’) aggregates, while initial dilution into buffer at 4 °C, followed by warming to 23 °C, results in formation of slow-growing aggregates (‘S-type’). (b) The formation of F-type and S-type aggregates was monitored in real-time using single angle, static light scattering at 340 nm. For reference, the observed light scattering from the native RuBisCO dimer is also shown. In every case, the final RuBisCO monomer concentration was 200 nM. The curves shown represent the average of n = 3 technical replicates with ensemble fractional uncertainties of 0.052, 0.039 and 0.017 for the F-type, S-type and native RuBisCO, respectively (see Supplemental Methods).


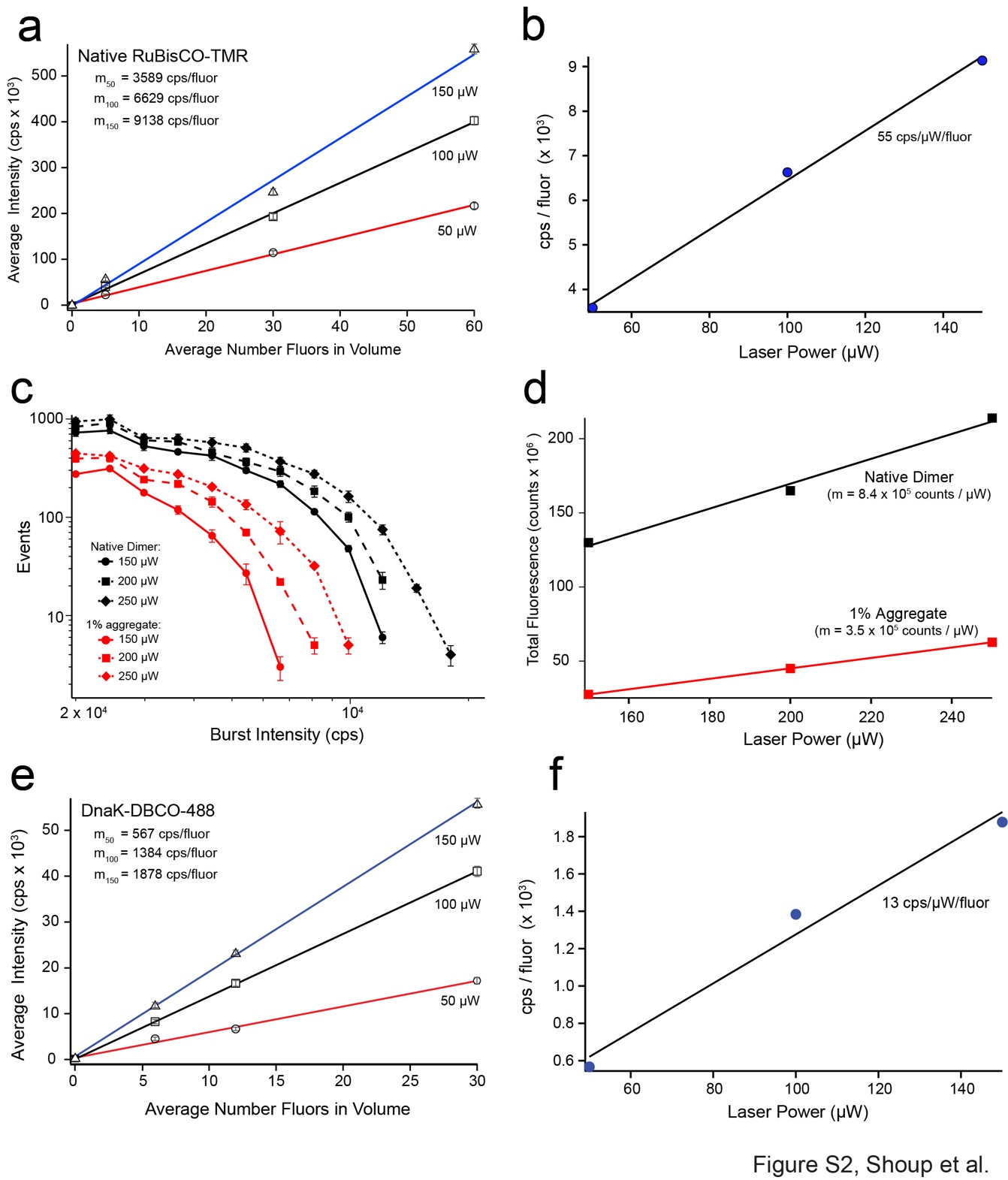


**Supplemental Figure 2. Calibration of the effective brightness of labeled RuBisCO and DnaK.** (a) The average fluorescence signal of the native RuBisCO-TMR dimer at different concentrations and excitation powers (561 nm) is shown. Error bars display the standard deviation of three independent technical replicates. (b) Dependence of the measured average fluorescence of the RuBisCO-TMR dimer on` excitation laser power. (c) Photon burst distribution of native single RuBisCO-TMR dimers and RuBisCO aggregate particles containing only one TMR-labeled monomer. Each sample (< 100 pM particles) was flowed through the BAS microscope confocal volume at a linear rate of 500 µm/sec. Aggregate particles containing a single labeled monomer were created with the same aggregation protocol described for F-type aggregates, except that only 1% of the input RuBisCO monomers carried an TMR dye. Samples were examined at three different laser powers and each curve represents the average of three independent experiments, with the error bars showing the standard deviation. Examination of the sample buffer alone resulted in less than a 2% contribution to the observed burst event number in only the first three, lowest amplitude data points (not shown). (d) Dependence of the total integrated fluorescence on laser power from the burst distributions shown in (c). (e) The average fluorescence signal of DnaK-DBCO488 in the presence of 1 mM ATP at different concentrations and excitation powers (488 nm) is shown. Error bars display the standard deviation of three independent technical replicates. (f) Dependence of the measured average fluorescence of the DnaK-DBCO488 on the excitation laser power.


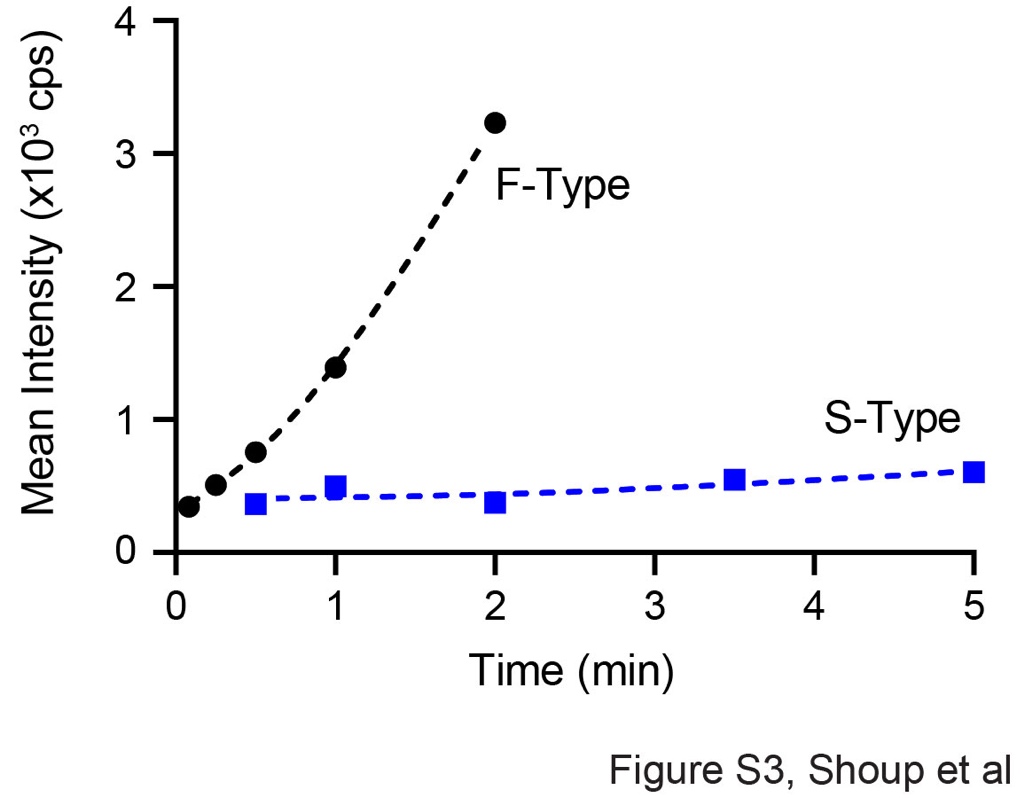


**Supplemental Figure 3. The average size of F-type particles increases much more rapidly than S-type particles.** The mean fluorescence burst amplitudes of the F-type and S-type particles, derived from Figure 1 e and f, are plotted as a function of time. Dashed lines show fits of the data to a spline function, which is intended as a visual guide.


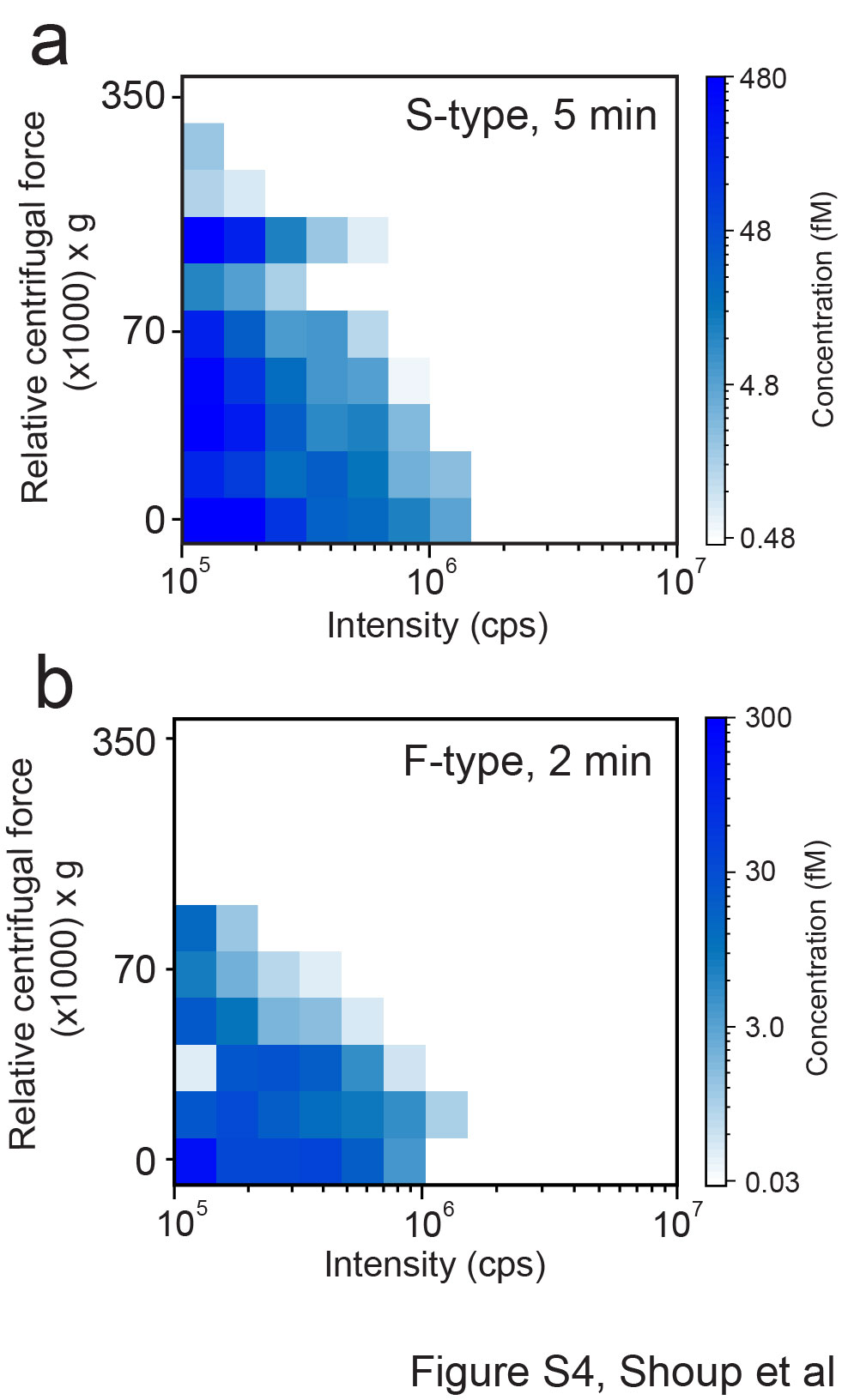


**Supplemental Figure 4. S-type** **and F-type** **aggregates display distinctive sedimentation behavior.** Following the initiation of aggregation, S-type (a) and F-type (b) RuBisCO-TMR aggregates were grown for the indicted times and then diluted to a final monomer concentration of 10 nM to halt aggregation. The samples were loaded into a TLA100.2 rotor of an Optima MAX-XP ultra-centrifuge (Beckman) and spun at increasing relative centrifugal force (rcf) for 10 min. At each indicated rcf, the supernatant was sampled and analyzed by BAS. The plots show the combination of n = 3 independent technical replicates.


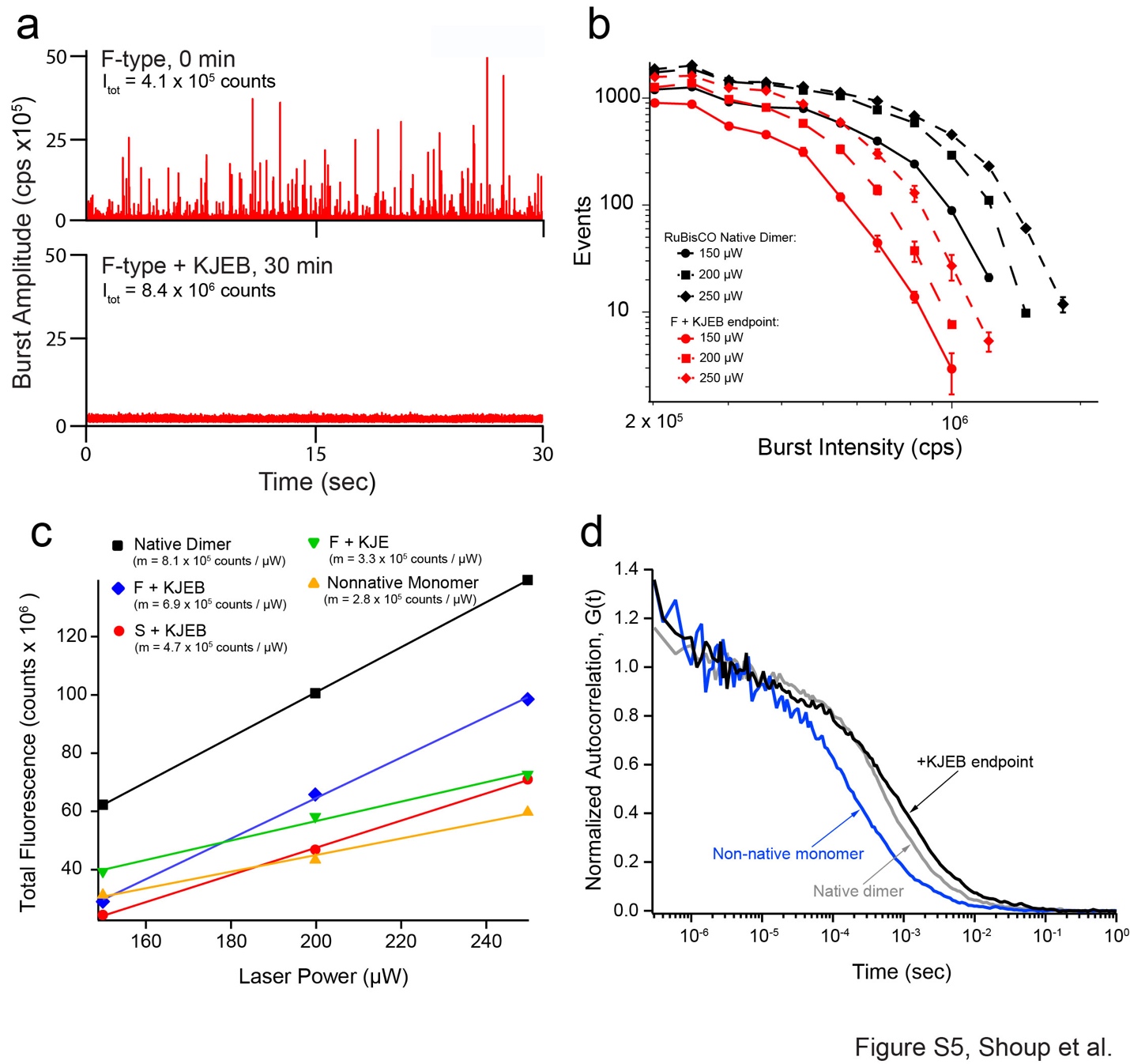


**Supplemental Figure 5. Disaggregation produces primarily monomeric RuBisCO that is most likely bound to DnaK and/or DnaJ.** (a) Raw fluorescence burst data for F-type (Fl) RuBisCO-TMR aggregates at 0 min and after 30 min of disassembly with KJEB (1 µM DnaK, 2 µM DnaJ, 2 µM GrpE and 200 nM ClpB, and 2 mM ATP, plus ATP regeneration). The integrated fluorescence intensity of each 30 sec photon history (I_tot_) is shown. (b) Photon burst distributions of single native RuBisCO-TMR dimers compared to 30 min disassembly endpoint in the presence of KJEB. Each sample was diluted to single molecule concentrations (< 50 pM labeled particles) and flowed through the confocal volume of the BAS microscope at a linear rate of 500 µm/sec. Samples were examined at three different excitation powers (561 nm laser) and each curve represents the average of three independent experiments, with the error bars displaying the standard deviation. The sample buffer contributed less than 2% to the observed burst event number in only the first three, lowest amplitude data points (not shown). Similar distributions were also generated for F + KJE, S + KJEB, and a nonnative monomer (not shown). (c) Each photon burst distribution was integrated and plotted as a function of laser power. (d) Normalized fluorescence correlation spectra (FCS) of a ~ 100 pM sample of native RuBisCO-TMR dimer, a non-native RuBisCO-TMR monomer, and the 30 min RuBisCO-TMR aggregate disassembly endpoint by KJEB.


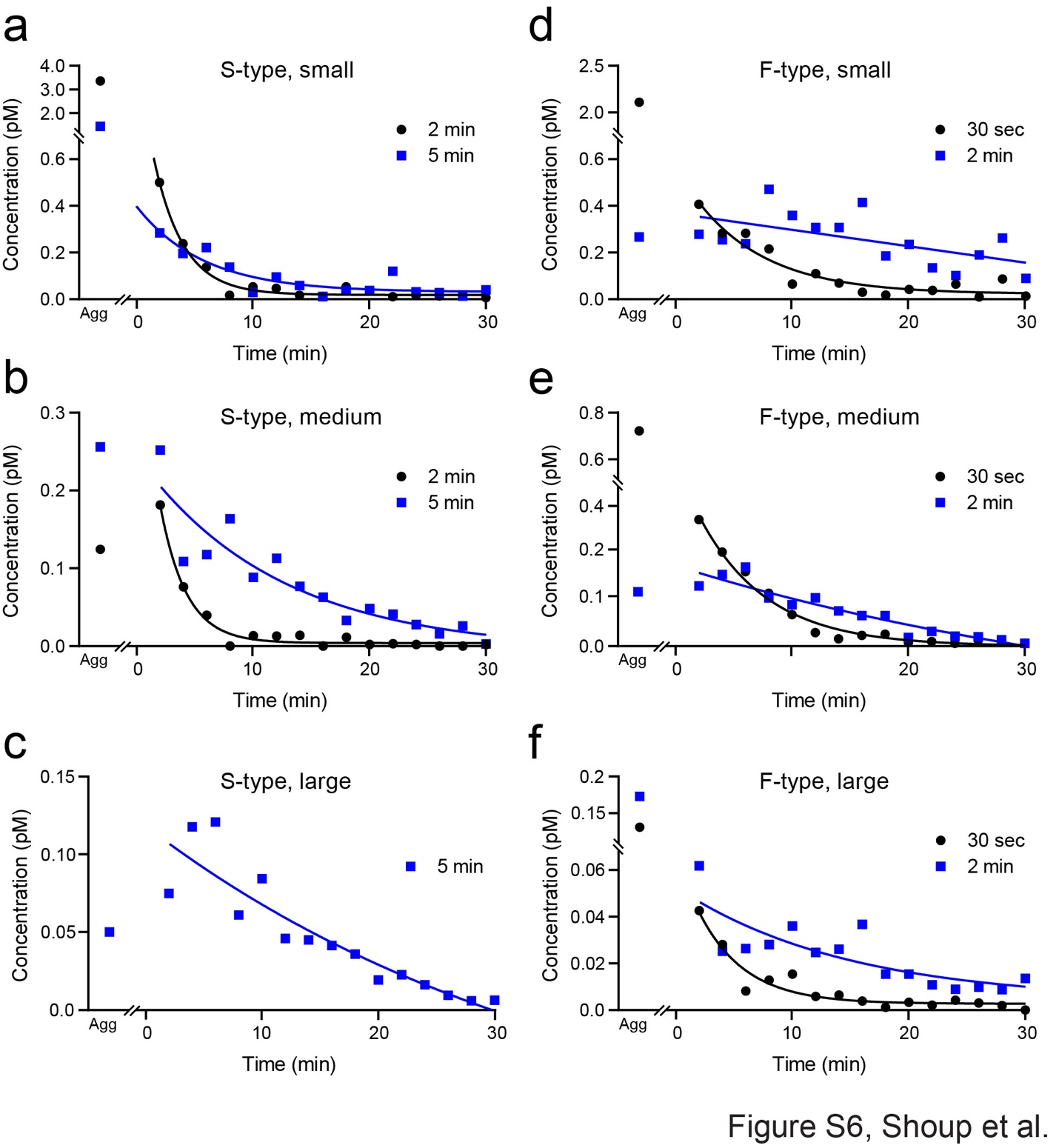


**Supplemental Figure 6. Disaggregation by KJEB slows for all particle sizes at later aggregation times.** BAS data from the population-resolved kinetics experiments shown in Figure 4 c-f was re-binned into three coarse size ranges: small (150-696 counts; ~ 40-200 mer), medium (696-3231 counts; ~ 200-900 mer) and large (3231-15,000 counts; ~ 900-4200 mer). The total particle concentration as a function of time in each coarse bin is shown for S-type (a-c) and F-type (d-f) aggregates at both earlier (2 min for S-type and 30 sec for F-type) and later (5 min for S-type and 2 min for F-type) aggregation time points. The starting concentration of aggregate particles in each coarse range, prior to addition of KJEB and sample loading, is also shown (*Agg*). No data for large particles is plotted for the early (30 sec) S-type aggregate sample, because this condition generates too few particles in this size range for analysis. To estimate the average disaggregation rate of particles in each coarse range, the data were fit to a single exponential rate law (solid lines). In each case, aggregate disassembly is slower when initiated from the later aggregation time points.


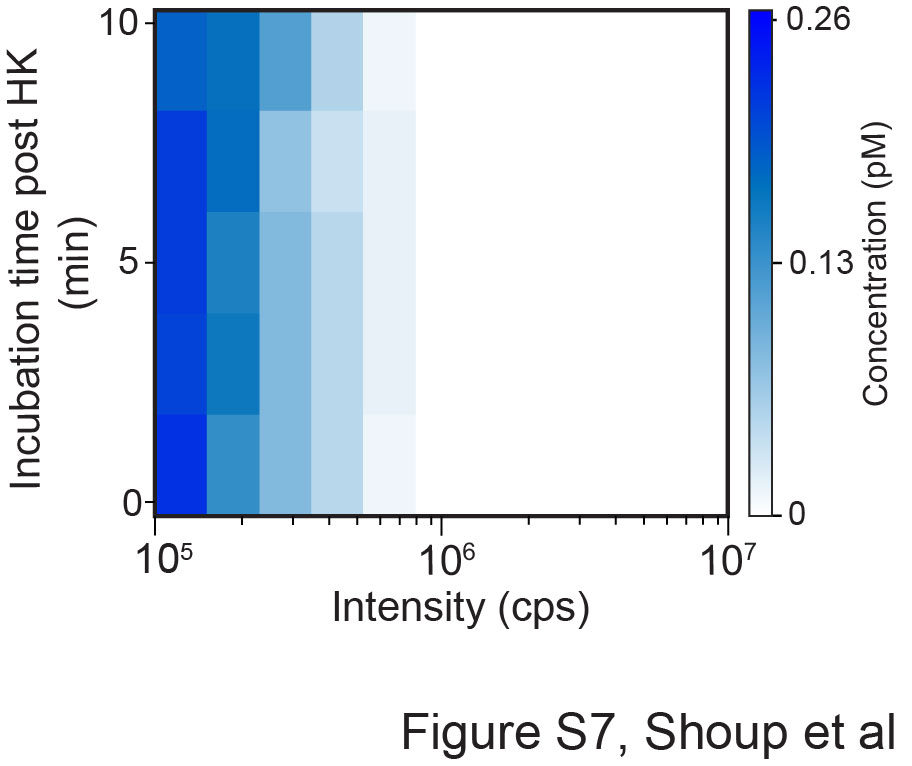


**Supplemental Figure 7. Depletion of ATP at intermediate disaggregating times does not result in re-aggregation.** S-type RuBisCO-TMR aggregates were grown for 3.5 min, then diluted 50-fold to a final monomer concentration of 10 nM in the presence of the KJEB system and ATP (0.5 µM DnaK, 1 µM DnaJ, 1 µM GrpE, and 300 nM ClpB, 2 mM ATP, and ATP regeneration) for 1 min. The sample was then rapidly depleted of ATP by addition of 0.05 U/µl of hexokinase and 20 mM glucose (Rye et al., 1997). Burst data was collected continuously for 10 min. The raw photon history was segmented into 2 min blocks and each block then subjected to BAS.


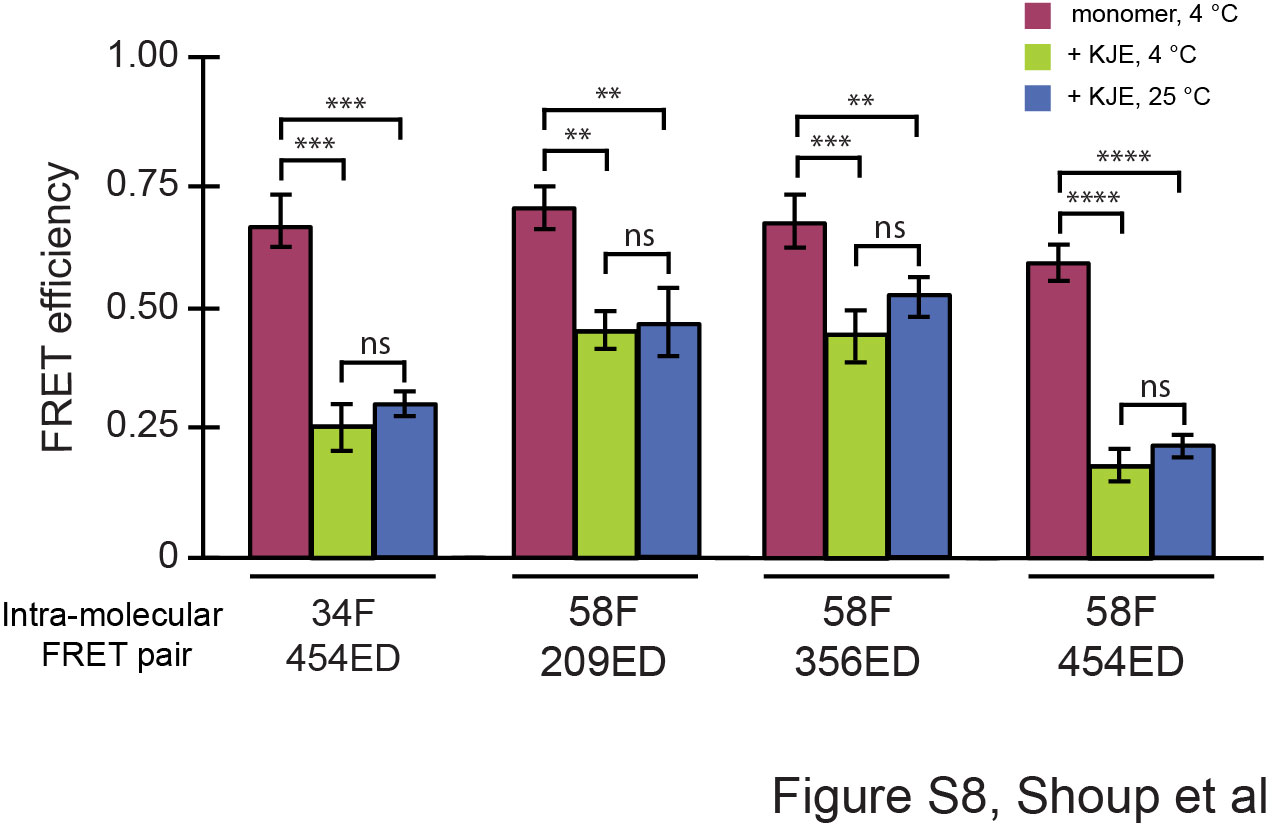


**Supplemental Figure 8. The KJE system imposes a multi-axis expansion on the non-native RuBisCO monomer.** The RuBisCO monomer that seeds S-type aggregates was examined by intra-molecular FRET following addition of the active KJE system. For each FRET pair, the kinetically trapped, non-native RuBisCO monomer was populated under non-aggregating conditions at 4 °C and was mixed with either buffer alone (monomer, *plum*) or with KJE and ATP (0.5 µM DnaK, 1 µM DnaJ, 1 µM GrpE, and 2 mM ATP; + KJE, *green*) at 4 °C. The final RuBisCO monomer concentration was 50 nM in each case. The average proximity of the donor- and acceptor-labeled segments, along four distinct spatial vectors, is inversely proportional to the observed donor-side, steady-state FRET efficiency (Lin and Rye, 2004; Lin et al., 2013). Samples mixed with KJE were also warmed to room temperature (25 °C) and the FRET efficiency re-determined (+KJE, *blue*). Each bar shows the mean and sd of n = 3 independent technical replicates. Significance was evaluated using a one-way ANOVA with P-values of ** < 0.01, *** < 0.001, and **** < 0.0001.

**SUPPLEMENTAL METHODS**

**Expression and purification of disaggregase molecular chaperones**

Expression of DnaK, DnaJ, and GrpE were all induced at an OD600 of 0.6, while ClpB expression was induced at an OD600 of 0.7. Cell cultures were lysed by high-pressure shear using a Model M-110Y Microfluidizer (Microfluidics) and lysates were then clarified using ultracentrifugation in a Ti-45 Beckman rotor at 35k RPM for 45 min. Similarly to previous work (Sweeny et al., 2011), proteins were first purified using consecutive Ni-NTA affinity (Quiagen) and Source 30Q (GE) ion exchange prior to His-tag removal using TEV protease. The proteins were then re-purified using using Ni-NTA and Source 30Q columns to remove both the cleaved tag and un-cleaved parent molecules. For DnaJ, a variation of this protocol was employed. After cell disruption, but before clarification by centrifugation, the cell lysate was mixed with de-ionized urea to a final concentration of 2 M. The supernatant from the centrifugation was then loaded onto a Ni-NTA affinity column, which was washed buffer containing 2 M urea in order to remove otherwise tightly bound, contaminating proteins that co-purified with DnaJ. Urea was then removed from the sample on-column using extensive washing in the same buffer without urea present.

**Static light scattering**

Static light scattering measurements were conducted with a PTI steady state fluorometer equipped with a thermally jacketed sample cuvette. Both excitation and emission monochromators were set to 340 nm and the slits of each optical path were narrowed to a bandpass of ≤ 1 nm. Formation of either F-type or S-type aggregates was first initiated as described in Methods at a final RuBisCO monomer concentration of 200 nM in a volume of 2.5 ml. Each sample was then rapidly transferred to cuvette pre-equilibrated at 23 °C. The light scattering signal at 90° was recorded continuously as a function of time. The effective dead-time between triggering of aggregation and initiation of data collection was approximately 10 sec for the F-type aggregates and 30 sec for the S-type aggregates. Average fractional uncertainties were calculated as

1. ${(\sum_{i=0}^{N} {\mathrm{SD}_{i}}/{{\langle y\rangle}_{i}})}/N$

where i is the time bin index, N is the number of data points in time, <y>_i_ is the mean light scattering value for the i-th bin averaged over the experimental repeats and SD_i_ is the standard deviation between experiment repeats for the i-th bin.

**Calibration of effective brightness of single fluorescent probes**

We first determined the average effective brightness of the TMR-maleimide dye coupled to a single, surface-exposed Cys residue at position 58 of the native RuBisCO dimer (Lin and Rye, 2004). The microscope effective confocal volume (V_eff_ ≈ 2 fL) was established using fluorescence correlation spectroscopy (FCS) measurements of known concentrations of a standard small dye. The average number of fluorophores in the probe volume was then determined from this V_eff_ in combination with known concentrations of the labeled RuBisCO dimers, established using a colorimetric Bradford assay calibrated with known concentrations of native RuBisCO (determined from total amino acid analysis). The average fluorescence of the labeled RuBisCO dimer was measured at three different laser powers between 50-150 µW (Figure S2a-b). The observed slopes of these lines were plotted as a function of input laser power in order to establish the effective average brightness of the TMR probe, yielding a value of 55 cps/µW/fluor. Similar analysis of DnaK-DBCO488 yielded an effective brightness of 13 cps/µW/fluor.

We next examined the effective brightness of the TMR dye upon incorporation into a RuBisCO aggregate. First, we measured the photon burst distribution of (1) single RuBisCO dimers, containing two dyes per dimer and (2) single aggregate particles containing no more than one labeled monomer per particle (Figure S2c-d). Each sample (< 100 pM particles) was flowed through the BAS microscope probe volume at a linear rate of 500 µm/sec. Aggregate particles containing single labeled monomers were created using the same basic aggregation protocol used for making F-type aggregates, except that only 1% of the input RuBisCO monomers carried a TMR dye, with the remainder unlabeled. Aggregation was halted by dilution after 1 min of growth. All samples were examined at three different excitation powers. The photon distribution curves display the expected amplitude roll off and the curves shift as a function of increasing laser power (Figure S2c). Integration of the observed photon distribution curves yields the total, confocal volume-weighted burst fluorescence (above background) for a TMR dye coupled to either the RuBisCO native dimer or a non-native monomer incorporated into an aggregate. The total fluorescence signal is then plotted as a function of input laser power in Figure S2d. The slopes of the lines in this plot are directly proportional to the effective brightness of the TMR dye in each context, and the ratio of the slopes can be used to determine whether aggregate incorporation alters the dye quantum yield (QY). If monomer incorporation into an aggregate had no impact on the TMR QY, the slope ratio in Figure S2d for the native dimer versus the singly labeled aggregates should be exactly 2. The observed slope ratio of 2.4 demonstrates that aggregate incorporation of a TMR-labeled RuBisCO monomer has, at most, a 20% average impact on the QY of the TMR dye.

**Analysis of RuBisCO disaggregation end points**

Three disaggregation conditions (F + KJEB, S + KJEB and F + KJE) resulted in the nearly complete disappearance of observable aggregate particles by 30 min at 23 °C. At the same time, as the observable aggregate particles disappeared, elevated concentrations of a much dimmer species appeared, which resulted in a rise in the mean fluorescence background over time (Figure S5a). We sought to characterize this disassembled population and determine whether it was composed of monomers or small oligomers. Disaggregation endpoint samples were first diluted to reduce the concentration of the TMR-labeled species to single molecule concentrations (< 50 pM). The fluorescence intensity distributions of the diluted endpoints were then examined as a function of laser power using a linear flow rate of 500 µm/sec (a representative data set for the F + KJEB condition is shown in Figure S5b). The burst distribution curves for all endpoint samples display amplitude roll offs and laser power dependencies consistent with species that possess an effective brightness that is lower than the native RuBisCO dimer. Integration of the observed photon distribution curves as a function of laser power yields the effective brightness of the TMR dye in each state (Figure S5c). For comparison, the effective brightness curves for the native RuBisCO dimer and a non-native monomer (Shoup et al., 2021) are also shown. The observed slopes are a function of (1) the number of TMR dyes per molecule and (2) the QY of the dye in each context. The non-native monomer appears to display a decrease in QY relative to the native dimer of ~ 30%. The effective brightness of the ‘F + KJE’ and ‘S + KJEB’ samples are consistent with these disassembly endpoints being dominated by monomeric RuBisCO. The effective brightness of the ‘F + KJEB’ is higher than would be expected for a purely monomeric species (Figure S5c), which could suggest a mixture of disassembled monomers and dimers. Alternately, the observed shift in effective brightness could be due to a monomeric RuBisCO species that displays an approximately 2x increase in TMR QY following complete disaggregation. A significant difference in brightness would suggest that, following disaggregation, the non-native RuBisCO monomer is either (1) conformationally distinct or (2) is bound differently by the DnaK and/or DnaJ chaperones. Intriguingly, this implies that the disaggregated RuBisCO does not populate a universal, non-native monomeric state. Rather the initial aggregation pathway and manner of disassembly (e.g., F + KJEB versus F + KJE) substantially influence the final properties of the released monomers.0
